# Kallistatin limits abdominal aortic aneurysm by attenuating generation of reactive oxygen species and apoptosis

**DOI:** 10.1038/s41598-021-97042-8

**Published:** 2021-08-31

**Authors:** Smriti Murali Krishna, Jiaze Li, Yutang Wang, Corey S. Moran, Alexandra Trollope, Pacific Huynh, Roby Jose, Erik Biros, Jianxing Ma, Jonathan Golledge

**Affiliations:** 1 The Vascular Biology Unit, Queensland Research Centre for Peripheral Vascular Disease, College of Medicine and Dentistry, James Cook University, Townsville, QLD 4811 Australia; 2 School of Applied and Biomedical Sciences, Faculty of Science and Technology, Federation University Australia, Horsham, VIC Australia; 3 Division of Anatomy, College of Medicine and Dentistry, James Cook University, Townsville, QLD Australia; 4 Department of Physiology, Health Sciences Centre, University of Oklahoma, Oklahoma City, OK 73104 USA; 5 Department of Vascular and Endovascular Surgery, Townsville University Hospital, Townsville, QLD Australia

**Keywords:** Cardiovascular biology, Cardiovascular diseases, Vascular diseases, Aneurysm

## Abstract

Inflammation, vascular smooth muscle cell apoptosis and oxidative stress are believed to play important roles in abdominal aortic aneurysm (AAA) pathogenesis. Human kallistatin (KAL; gene *SERPINA4*) is a serine proteinase inhibitor previously shown to inhibit inflammation, apoptosis and oxidative stress. The aim of this study was to investigate the role of KAL in AAA through studies in experimental mouse models and patients. Serum KAL concentration was negatively associated with the diagnosis and growth of human AAA. Transgenic overexpression of the human *KAL* gene (*KS-Tg*) or administration of recombinant human KAL (rhKAL) inhibited AAA in the calcium phosphate (CaPO_4_) and subcutaneous angiotensin II (AngII) infusion mouse models. Upregulation of KAL in both models resulted in reduction in the severity of aortic elastin degradation, reduced markers of oxidative stress and less vascular smooth muscle apoptosis within the aorta. Administration of rhKAL to vascular smooth muscle cells incubated in the presence of AngII or in human AAA thrombus-conditioned media reduced apoptosis and downregulated markers of oxidative stress. These effects of KAL were associated with upregulation of Sirtuin 1 activity within the aortas of both *KS-Tg* mice and rodents receiving rhKAL. These results suggest KAL-Sirtuin 1 signalling limits aortic wall remodelling and aneurysm development through reductions in oxidative stress and vascular smooth muscle cell apoptosis. Upregulating KAL may be a novel therapeutic strategy for AAA.

## Introduction

Abdominal aortic aneurysm (AAA) is an important cause of mortality^1^. Current guidelines recommend surgical repair of large (maximum aortic diameter ≥ 55 mm in men and ≥ 50 mm in women) asymptomatic or symptomatic AAAs and imaging surveillance alone for small asymptomatic AAAs (< 55 mm in men and < 50 mm in women)^2^. The increasing use of abdominal imaging and ultrasound screening programs means that many AAAs are identified when they are small and asymptomatic^3^. Up to 70% of small AAAs grow to a size at which surgical repair is indicated^4,5^. The discovery of drug therapies effective in limiting AAA growth would markedly change current patient management^5–8^.

Human kallistatin (KAL; gene *SERPINA4*) is a member of the serine proteinase inhibitor superfamily^9^. KAL is expressed in circulating and aortic cells including vascular smooth muscle cells (VSMCs) and therefore could theoretically play a protective role against human AAA formation and progression^10^. Recent research suggests that the Wingless integrated (Wnt) pathway promotes AAA pathogenesis, while the Sirtuin 1 (SIRT1) pathway inhibits AAA formation^11,12^. KAL has been reported to be an endogenous inhibitor of the Wnt pathway and to activate the SIRT1 pathway^13,14^. Furthermore, previous animal and in vitro studies suggest that KAL inhibits reactive oxygen species (ROS) formation and limits inflammation and extracellular matrix (ECM) remodelling and blocks cellular apoptosis, which are all implicated in AAA pathogenesis^8^. Recent research also suggests that KAL inhibits atherosclerosis development in dyslipidemic mice^15^.

In view of this previous research, it was hypothesised that KAL would inhibit AAA pathogenesis. This theory was studied using patient samples and mouse models. Specifically it was hypothesised that circulating concentrations of KAL would be negatively associated with human AAA diagnosis and growth. It was also hypothesised that upregulation of KAL would reduce the severity of AAA in two mouse models of AAA.

## Methods

### Study design

This study was designed to carry out the following aims: (1) to examine the association of circulating KAL concentrations with AAA diagnosis and growth in patients; (2) to examine the effect of administering recombinant human (rhKAL) on AAA development in a mouse model; (3) to examine the effect of rhKAL on human aortic VSMCs in vitro and (4) to examine the effect of overexpressing human KAL on AAA development within a mouse model.

### Human blood and tissue samples

Serum samples were obtained from men involved in the Health in Men Study (HIMS), which has been previously described in detail^16^. Community-dwelling men (n = 12,203) aged 65–83 years took part in a randomised trial of ultrasound screening for AAA. Each man had an abdominal ultrasound between 1996 and 1999 (HIMS wave 1). In 2001–2004, (HIMS wave 2) blood samples were collected from 4249 of the men. For the current study, men (n = 304) who were found to have an AAA on ultrasound [infra-renal aortic diameter (IRA) ≥ 30 mm] and which serum samples were available were included. We randomly selected approximately twice as many men (n = 652) in which no AAA was identified (IRA diameter < 30 mm) as controls. The Human Research Ethics Committee of the University of Western Australia approved the protocol for HIMS (RA/4/1/5765) and all men provided written informed consent.

A further group of patients with large AAAs undergoing open repair were recruited from Townsville University Hospital, Australia. Full thickness samples were obtained from the anterior wall of the aneurysm body (site of maximum aortic dilation; AAA-Body, n = 12) and macroscopically non-dilated proximal neck of aneurysm (site immediately distal to the renal arteries where the aortic diameter was relatively normal; AAA-Neck, n = 6) using procedures previously described^17^. The study was approved by the Human Ethics Committees of the Townsville Hospital and Health Services and James Cook University (HREC/12/QTHS/202). All protocols conformed to the ethical guidelines of the Declaration of Helsinki and samples were obtained after receiving written informed consent.

### Risk factors

These were collected by assessment of medical records and interview. Waist and hip circumference were measured in accordance with guidelines of the International Society for the Advancement of Kinanthropometry^18^. Diabetes and hypertension were defined by a history of diagnosis or treatment of these conditions. Coronary heart disease (CHD) was defined by a history of myocardial infarction, angina or treatment for coronary artery disease. Previous stroke was defined by a previous history of any stroke. Smoking was defined by history as ever having smoked regularly or never having smoked regularly. Prescribed medications were also recorded.

### Aortic imaging

Aortic ultrasound was performed as previously described^16^. The greatest diameter of the IRA was measured from the outer to the outer wall of the aorta using a Toshiba Capasee ultrasound machine with a 3.75 MHz probe (Toshiba Australia, North Ryde, NSW)^19^. In participants in which a small AAA was detected, repeat aortic imaging was recommended at intervals of 6 months if the initial diameter was ≥ 40 mm and 12 months if the initial diameter was 30–39 mm. The reproducibility of ultrasound measurements was assessed every 4 months by repeat scanning of 10 randomly selected participants on two occasions by the three different sonographers. No significant differences were found between observers, with 95% of differences in each of antero-posterior and transverse diameters being < 3 mm^19^.

### Blood assays

Serum KAL was measured with a commercial assay (R&D Systems, Minneapolis, USA) by an experienced scientist. The inter-assay coefficient of variation was 5%. Serum creatinine, low density lipoprotein (LDL) and high density lipoprotein (HDL) were measured using automated assays (Hitachi 917, Roche Diagnostics GmBH, Mannheim, Germany), as previously described^20^. The inter-assay coefficient of variation for these assays was between 2 and 5%. Serum high sensitivity C-reactive protein (hs-CRP) was measured by a high-sensitivity assay, with the use of the particle-enhanced immunonephelometry system on the BNII analyser. The inter-assay coefficient of variation was 4–7% (Dade Behring)^19^.

### Mouse models and in vivo studies

The mouse studies were performed in accordance with institutional and ethical guidelines of James Cook University, Australia. The experimental protocols were in accordance with the ARRIVE guidelines and were approved by the James Cook University animal ethics committee (AEC approval A2125). All animal protocols conformed to the Guide for the Care and use of Laboratory Animals (National Institutes of Health, United States) and the Australian Code of Practice for the Care and Use of Animals for Scientific Purpose (7th Edition, 2004) and the criteria set by The National Centre for the Replacement, Refinement and Reduction of Animals in Research (London, UK). All mice were kept under 12 h day/night light cycle with the temperature in the animal facility maintained at 23 ± 2 °C and the relative humidity of 55 ± 2%. The mice were maintained on normal laboratory chow and given water ad libitum. All procedures were performed under sterile conditions with surgical magnification. Mice were humanely euthanized at the end of the study using CO_2_ asphyxiation. On completion of an experiment, blood was collected by cardiac puncture and the aorta was harvested from the aortic arch to the femoral bifurcation, perfused with phosphate buffered saline (PBS) and processed for subsequent ex vivo morphometry measurements. Approximately 1 mm aortic segments immediately below (CaPO_4_ model) or above (AngII model) the renal arteries were then harvested. For consistency, the segment close to renal branches was processed for histopathology assessment and the distal segment was processed for RNA extraction.

Two different animal models of AAA were used to examine the effect of KAL on AAA:

#### Calcium phosphate (CaPO_4_) mouse model of AAA

This model exhibits aortic dilation associated with inflammation and apoptosis^21,22^. This model was used to examine the effect of transgenic overexpression of the human *KAL* gene (*KS-Tg*) on AAA development. The CaPO_4_ model was preferred because the *KS-Tg* mice were on a genetic background of the C57BL/6 strain which is resistant to aneurysm induction by angiotensin II (AngII) infusion^13^. KAL overexpression was confirmed by genotyping and protein and mRNA expression assessments (Supplementary Fig. S1). Control mice were littermate WT controls, not expressing the KAL transgene. For AAA induction, mice were anaesthetised by isoflurane inhalation in conjunction with oxygen during surgery (4% isoflurane/O_2_ inhaled) according to a previously published protocol^21^. A midline abdominal incision was made and the IRA exposed. A small piece of filter paper (5 mm × 5 mm) pre-soaked in 0.5 mol/l calcium chloride was affixed to the exterior wall of IRA for 10 min and then removed. A second small piece of filter paper of similar size pre-soaked in PBS was then affixed to the same part of the exterior wall of IRA for 5 min and then removed. After closing the abdominal wound with sutures, the mouse was allowed to recover under an infrared heating lamp until it regained normal physical activity.

#### Ang II mouse model of AAA

Aneurysms in this model have marked upregulation of inflammatory markers similar to human AAA^23,24^. Administration of recombinant KAL (rhKAL) is a feasible means to upregulate KAL in vivo. In a preliminary study injection of 21.6 µg/kg of rhKAL i.p. led to a measurable level of circulating KAL after one hour (plasma KAL approximately 1.4 ng/ml). This dose of rhKAL was therefore chosen for further investigation. AngII was infusion subcutaneously into apolipoprotein E deficient (*ApoE*^−/−^) mice at a rate of 1.0 µg/kg/min over 28 days as previously described^11^. Osmotic micro-pumps (ALZET Model 1004, Durect Corporation, USA) were inserted into the subcutaneous space left of the dorsal midline under anaesthesia (4% isoflurane/O_2_ inhaled) to administer AngII. Male *ApoE*^−/−^ mice (n = 40) were randomly allocated to receive recombinant KAL (rhKAL, R&D Systems, 1669-PI, 21.6 µg/kg/d i.p.) or saline vehicle control (VC) during the 4 week AngII-infusion.

Mice in both experiments were monitored for 28 days. Systolic blood pressure was measured by tail cuffs using the CODA machine (Kent Scientific Corporation) at baseline, 14 and 28 days, as reported previously^11^.

### Assessment of AAA severity

Maximum aortic diameter was measured in vivo using transabdominal ultrasonography during the experiments and by ex vivo morphometry assessment at completion of the study.

Ultrasound measurements of the IRA or SRA were obtained at baseline and at days 14 and 28. Scans were performed on anesthetised mice (i.p. 40 mg/kg ketamine and 4 mg/kg xylazine) using a MyLabTM 70 VETXV machine (Esaote, Italy) with a 40 mm linear transducer at an operating frequency of 10 MHz (LA435; Esaote, Italy) to provide an external sagittal image of the aortic segment assessed. Maximum IRA or SRA diameter was measured at peak systole in the anterior to posterior plane from the outer-to-outer aortic wall, using the caliper measurement feature as previously reported and as has been shown to be highly reproducibility^11^.

Ex vivo morphometry measurements were performed on aortas harvested from mice that completed the full 28 day experiments. Immediately following euthanasia, the aortas were perfused with PBS under constant physiological pressure, harvested, placed on a graduated template and digitally photographed (Coolpix 4500, Nikon). Maximum external diameters of the aortic arch, thoracic aorta, IRA and SRA were determined from the images using computer-aided analysis (Adobe Photoshop CS5 Extended, *version 12*, Adobe Systems Incorporated). These measurements have been shown to be highly reproducible^11^.

The left and right carotid arteries and aortic arch were cut opened longitudinally and secured with pins on a wax-coated petri-dish and photographed (Coolpix 4500, Nikon). The area of the ascending aorta was quantified by measurement of the intimal area of the region from the aortic origin to 3 mm proximal to the subclavian artery (Adobe Photoshop CS5 Extended, *version 12*, Adobe Systems Incorporated) following a previously published protocol^25^.

### Quantification of atherosclerotic lesion area

The severity of atherosclerosis was quantified through examination of the intimal surface of the ascending aorta by *en face* Sudan IV staining as previously described^26^. The aortic tissue samples were transferred from PBS to a 70% ethanol solution and stained with 0.1% Sudan IV dissolved in equal parts of acetone and 70% ethanol for 10 min. Background staining was washed off by 10 min incubation in 70% ethanol. Samples were rehydrated by brief placement in H_2_O and digitally photographed in order to identify areas of staining (Coolpix 4500, Nikon). Sudan IV stained areas were quantified using Adobe Photoshop software (*version CS5*). The atherosclerotic lesion area was estimated as a percentage of the total luminal surface area stained, as previously described^26^.

### Assessment of plasma cytokine levels

Platelet poor plasma samples were examined by a multiple analyte mouse inflammation cytometric bead array (CBA, BD Biosciences) to quantitatively concentrations of interleukin (IL)-6, IL-10, monocyte chemoattractant protein-1 (MCP-1), interferon-gamma (IFN)-γ, tumor necrosis factor (TNF)-α and IL-12p70 according to the manufacturer’s instructions. Flow cytometry was performed on a CyAn ADP flow cytometer (Beckman Coulter) and results were analysed with FCAP Array software (*v3.0*, BD Biosciences), following previously published protocols^21^. This method has a coefficient of variation of 6–9%^21^.

### Histopathological assessments

IRA or SRA segments (n = 6–8/group) stored in O.C.T. compound, from each experimental group were selected using a random number generator. 6 µm thick sections were serially cut from the aorta immediately below (CaPO_4_ model) or above (AngII model) the renal arteries. Adjacent sections. (3–4 sections/sample) were stained with Hematoxylin and eosin (H&E, ProSciTech) and elastin van Gieson (EVG; Polysciences, Inc). The sections were photographed using a Nikon Eclipse 50*i* microscope fitted with a CCD Camera (DSF*i*1) and digital images captured (NIS Elements *vF2.30*). Elastin filament fragmentation was assessed under 400× magnifications from 2 to 3 aortic sections from each mouse and was graded as follows: 1—no elastin breaks, 2—mild breaks; 3—moderate to severe breaks; and 4—severe elastin filament fragmentation. These assessments can be performed with good inter-observer reproducibility^21^.

For collagen staining, serial cryostat sections (6 µm thickness) were fixed in 80% methanol at – 20 °C before rehydration in distilled water. Sections were then placed in Weigerts hematoxylin (ProSciTech) followed by rinsing in distilled water until the water was clear. Sections were then placed in Picro Sirius Red solution (ProSciTech) for 60 min followed by quickly removing excess Picro Sirus Red in 70% ethanol. Sections were then dehydrated in serial ethanol solutions and two xylene solutions. Images of stained sections were taken under a light microscope using reflective transmitted light and a circular polarising filter (Zeiss Axiovision). Sections were photographed with identical exposure setting for each section. Images were analysed in Adobe Photoshop CS6 software using the area measurement tool to calculate collagen content percentage in the total section area. The percentage of birefringence was determined for 4–5 fields for at least three sections per mouse and the mean value was calculated for each region as described previously^11^. These assessments are highly reproducible^11^.

### TUNEL staining

In situ terminal deoxynucleotidyl transferase mediated dUTP nick-end labelling (TUNEL) was performed using an in situ apoptosis kit (VasoTACS, Trevigen) to localize cells undergoing nuclear DNA fragmentation. Cryostat sections (6 µm) from the IRA and SRA were processed for the TUNEL assay as described previously^26^ and both positive and negative control slides were included (n = 6/group). The presence of apoptotic cells was identified as dark nuclear staining with the distinctive morphological appearance associated with cell shrinkage and chromatin condensation (apoptotic cells) or cytoplasmic fragments with or without condensed chromatin (apoptotic bodies). The images were captured at 400× magnification and percentage of positive staining area was calculated by dividing the positive staining area by the total area of the region selected. For each region of interest, a spot check was performed by visually counting the cells and avoiding necrotic areas. All histological evaluations were done in a blinded fashion in at least 3–4 sections per sample. These assessments can be performed with good reproducibility (Intra-assay coefficient of variation = 3%)^26^.

### In situ imaging of superoxide

Oxidative stress was estimated in unfixed frozen tissue sections from O.C.T. embedded IRA and SRA segments stored at – 80 °C using dihydroethidium staining (DHE, Sigma-Aldrich) according to a previously published protocol^27^. Briefly, cryosections (6 µm, n = 6/group) were brought to room temperature for 5 min. Slides were covered with Hanks' Balanced Salt solution (HBSS, Sigma-Aldrich) for 5 min, then DHE solution in HBSS was added to sections for 20 min at room temperature in a dark, humidified chamber. Slides were transferred to a 37 °C incubator for 30 min. Slides were washed three times with HBSS, washed with PBS and mounted on coverslip using antifade mounting medium (VECTASHIELD, Vector Labs). Fluorescent images were captured with a microscope and scored for red fluorescence intensity by an observer blinded to the experimental group (580-nm filter, Zeiss Axiovision). Quantitative measurements of the DHE fluorescence intensity were performed using the inbuilt software (Zeiss Axiovision). The average pixel intensities were calculated and reported as DHE intensity Arbitrary Units (A.U.).

### SIRT1 deacetylase activity assay

Nuclear proteins were extracted from randomly selected IRA and SRA segments using a nuclear extraction kit (Abcam) according to the manufacturer’s instructions. The SIRT1 activity was assayed using a SIRT1 activity assay kit (Abcam) according to the manufacturer’s instructions. The kit measures the activity of SIRT1 based on fluorescence intensity using a microplate reader with an excitation wavelength of 350 nm and an emission wavelength of 460 nm (BMG Labtech). The measured fluorescence is directly proportional to the deacetylation activity of the enzyme within the sample and results were presented as relative fluorescence units (RFU)/μg protein.

### Cell culture studies

Commercially available healthy human aortic VSMCs (CC-2571, Lonza) were serially passaged using trypsin/EDTA (Sigma Aldrich) and used for experiments between passages three and six. For some VSMC experiments, cells were incubated with conditioned medium obtained from AAA intra-luminal thrombus (ILT) explant cultures. The preparation of ILT-conditioned media was as previously published^11^. Briefly, ILT tissues were rinsed and sliced finely in sterile PBS (pH 7.4) and incubated in DMEM [supplemented with penicillin, streptomycin, glutamine, 5% (*vol/vol*) FBS and 2 µg/ml Fungizone] at 37 °C in a humidified 5% CO_2_ atmosphere. The culture media was refreshed after 1 h and the explants were cultured for a further 20.5 h to provide ILT-conditioned media. The thrombus conditioned media was decanted and centrifuged at 5000×*g* for 10 min to pellet large debris and the supernatants containing secreted thrombus proteins were snap frozen and stored at – 80 °C. Cells (1 × 10^6^ cells/ml) were plated out in 12 well culture plates and incubated with 5% DMEM (Gibco). After settling overnight, cultures were replenished with fresh media that contained ILT-conditioned media for 24 h and media removed and replenished with fresh media plus 10 nM rhKAL (1669-PI, R&D Systems) or varying doses of fenofibrate for an additional 24 h. The control group received a fresh media change at the same time. For AAA-Body and AAA-Neck VSMCs, all experiments were conducted using cells at passage three (Supplementary Table S3). At the end of experiment period, supernatant was removed and cells harvested for both protein and RNA isolation. For protein extraction, cells were harvested in RIPA buffer (Cell Signalling Technology) containing protease inhibitors (Roche) and phosphatase inhibitors (Pho-STOP, Roche). Protein concentration was determined by using the BioRad protein assay (Bio-Rad Laboratories) and SERPINA4 concentration levels were assessed (#CSB-EL021060HU, CUSABIO) according to the manufacturer's recommendations. Cells were collected into RNAlater (Ambion) for RNA isolation using RNeasy Mini kit following the manufacturer’s protocol (Qiagen). All experiments were performed in triplicate.

### Assessment of apoptosis in vitro

Caspase-3 activity was measured in homogenates of VSMCs using the Caspase-Glo 3/7 assay kit (Promega) according to the manufacturer's instructions. Luminescence activity was measured within 30–60 min, in six separate experiments using the Polar Star Omega (BMG Labtech).

### Assessment of oxidative stress in vitro

VSMCs were plated on opaque, multiwell plates and incubated with ILT-conditioned media and rhKAL as explained in the previous sections. At the end of the experiment period, the ROS-Glo™ H_2_O_2_ substrate (Promega) was incubated directly with cells in culture media to generate a luciferin precursor. Addition of ROS-Glo™ Detection Solution converted the precursor to luciferin and produced a light signal that is proportional to the level of H_2_O_2_ present in the sample. Luminescence activity was measured within 30 min, in six separate experiments using the Polar Star Omega (BMG Labtech).

### mRNA analysis by quantitative real-time PCR

Total RNA was isolated from IRA or SRA segments, human AAA tissues and VSMCs. At the time of harvest a segment of IRA or SRA was stored in RNA Later (Qiagen). Aortas were selected using a random number generator for the gene expression analysis (n = 8/group) and initial tissue digestion was processed using a bullet blender with stainless beads to maximise the yield. For RNA isolation from cell culture experiments, cells were harvested after experiments in Trizol (Qiagen). Total RNA was isolated using an RNeasy Mini kit (Qiagen) according to manufacturer’s instructions and quantified spectrophotometrically using the Nanodrop 2000. RNA samples were subjected to quantitative real time PCR (QRT-RCR) analysis of genes of interest using the QuantiTect SYBR Green one-step RT-PCR assay (Qiagen). QRT-PCR was performed to evaluate gene expressions using primers for human *SERPINA4, SIRT1,* Matrix metalloproteinase-9 (*MMP-9*), *MMP-2,* vascular endothelial growth factor (*VEGF*) and Glyceraldehyde 3-phosphate dehydrogenase (*GAPDH*) and mouse *Mmp-9*, *Mmp-2*, *Vegf*, Osteoprotegerin *(Opg),* Osteopontin (*Opn)* and *Gapdh* (Qiagen). Primers are listed in Supplementary Table S4. The relative expression of these genes was calculated by using the concentration-Ct-standard curve method and normalized using the average expression of glyceraldehyde-3-phosphate dehydrogenase (human: *GAPDH*, QT00079247; mouse: *Gapdh*; QT01658692) for each sample using the Rotor-Gene Q operating software (*version 2.0.24*).

### Statistical analysis

For human studies, quantitative data were not normally distributed and therefore were presented as median and inter-quartile range (IQR) and compared between groups using appropriate non-parametric tests including Mann Whitney *U* and Kruskal–Wallis tests. Nominal data were presented as number and percentages and compared by Chi-squared test. Men were divided into quartiles based on serum KAL. The association of serum KAL with AAA diagnosis was assessed using multiple logistic regression analysis adjusting for age, CHD, hypertension, diabetes, smoking, stroke, waist-to-hip ratio (WHR), serum creatinine, HDL, LDL and aspirin prescription. In men with repeat ultrasound imaging, change in AAA diameter was calculated by averaging the change between successive ultrasound scans and adjusting for the observation time as previously described^28^. Since average weighted yearly change in AAA diameter was associated with the initial AAA diameter^29^, the association between serum KAL and average AAA growth per year was also expressed as a percentage of the initial AAA diameter. Furthermore, the association of serum KAL quartile with AAA growth greater than median was analysed by logistic regression, adjusting for other risk factors, including initial AAA diameter, diabetes and serum CRP. The relationship between serum KAL and AAA growth was also assessed using linear regression adjusting for the same risk factors.

For mouse studies, the normality of the data was tested using the D’Agostino and Pearson omnibus normality test. Results are expressed as median and interquartile range (IQR) for non-normally distributed data and as mean ± standard error of mean (S.E.M) for normally distributed data. Normally distributed data were compared using t test or ANOVA. Comparison for non-normally distributed data were made using Mann–Whitney *U*-test or Kruskal–Wallis test, where appropriate. The change of aorta diameter over time measured with ultrasound and blood pressure measurements during the experimental period were assessed using repeated measures 2-way ANOVA. Kaplan–Meier survival curves were analysed using log-rank (Mantel-Cox) test. Data was analysed using SPSS software (*version 22*) or by GraphPad Prism (*version 7*). In all cases, *P* values <0.05 were considered significant.

## Results

### Serum KAL was negatively associated with AAA diagnosis

The risk factors of the 956 men included in relation to whether they had an AAA diagnosed or not are shown in Supplementary Table S1. Serum KAL concentrations were significantly lower in men diagnosed with an AAA. Serum KAL quartiles were incrementally associated with reducing risk of having an AAA after adjusting for other risk factors (Table 1). Men with serum KAL concentrations in the second, third and fourth quartiles had a 0.50 (95% confidence intervals, CI 0.33–0.75), 0.47 (95% CI 0.31–0.71) and 0.31 (95% CI 0.20–0.48) odds ratio of having an AAA diagnosed, respectively, by comparison to men with serum KAL concentrations in the first quartile. An increase of 6 ng/ml of serum KAL concentration (approximately standard deviation) was associated with a 0.73 odds reduction (95% CI 0.62–0.86) of having an AAA diagnosed after adjusting for other risk factors (Table 1).

**Table 1:** The independent association of serum KAL quartiles with AAA diagnosis assessed using multiple logistic regression in samples from 956 men.

Characteristic	Odds ratio	95% CI	*P* value
**Model 1**			
Serum KAL per 6 ng/ml*	0.73	0.62–0.86	< 0.001
**Model 2**			
Serum KAL in ng/ml^a^			
< 13.15	1	Reference	
13.15–16.29	0.50	0.33–0.75	0.001
16.30–20.30	0.47	0.31–0.71	< 0.001
> 20.30	0.31	0.20–0.48	< 0.001

Both models were adjusted for hypertension, diabetes, ever smoking, coronary heart disease, past history of stroke, waist to hip ratio, serum creatinine, serum low density lipoprotein, serum high density lipoprotein and C-reactive protein.

AAA, Abdominal aortic aneurysm; CI, confidence interval; KAL, kallistatin.

*Approximate standard deviation of KAL.

^a^Men with serum KAL concentrations in the top, third and second quartiles were compared with subjects who had serum KAL in the lowest quartile.

### Serum KAL was negatively associated with AAA growth

Men with AAA (n = 272) measuring a median (IQR) initial IRA diameter of 32.9 mm (31.0–36.1) were followed for a median (IQR) of 5.5 years (5.0–6.0) with a median of 7 (6–7) ultrasound scans. Median (IQR) annual increase in maximum AAA diameter (AAA growth) was 1.2 mm/year (0.5–1.9). AAA growth was negatively correlated with serum KAL concentration (Spearman’s rho − 0.173, *p* = 0.004; Supplementary Fig. S2). Men with serum KAL in the lowest quartile had significantly greater rate of AAA growth whether assessed in mm increase/year or percentage of initial diameter increase (Supplementary Table S2). Serum KAL was negatively associated with AAA growth after adjusting for initial AAA diameter, diabetes and serum high sensitive C-reactive protein (hs-CRP) using linear regression (Beta − 0.118, *p* = 0.036) and logistic regression (Table 2).

**Table 2:** Independent risk factors for AAA growth greater than median in 272 men.

Characteristic	Odds ratio	95% CI	*P* value
Serum KAL in ng/ml*			
< 11.96	1	Reference	
11.96–14.85	0.46	0.22–0.96	0.038
14.86–18.00	0.36	0.17–0.76	0.007
> 18.00	0.49	0.24–1.04	0.062
Initial AAA diameter > 32.90 mm^a^	2.99	1.78–5.01	< 0.001
Diabetes mellitus	0.27	0.11–0.67	0.005
Serum hs-CRP > 2.46 mg/ml^a^	0.81	0.48–1.37	0.444

For nominal variables, the comparisons are to subjects without the risk factor.

AAA, Abdominal aortic aneurysm; CI, confidence interval; hs-CRP, high sensitivity C-reactive protein; KAL, kallistatin.

*Men with serum KAL concentrations in the top, third and second quartiles were compared with subjects who had serum KAL in the lowest quartile.

^a^Approximately median value.

### Transgenic overexpression of human KAL in mice attenuated CaPO_4_-induced aortic expansion

**Figure 1:**
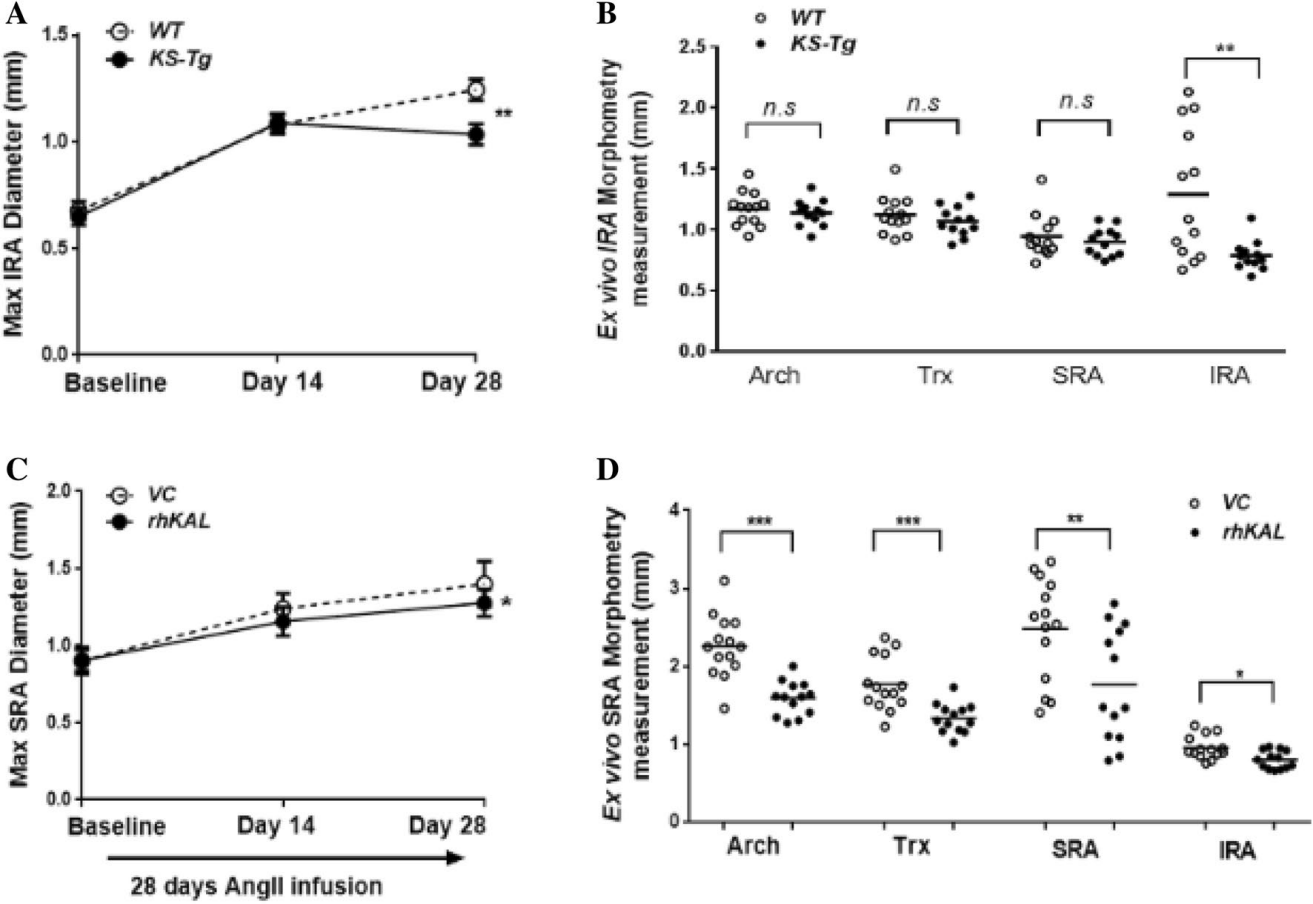
The effect of human KAL upregulation in AAA mouse models. (**A, B**). Effect of transgenic overexpression of KAL on CaPO_4_-induced aortic dilatation. (**A**) Shown are maximum external IRA diameter of *WT* (n = 13) and *Kal-Tg* (n = 12) mice both subjected to peri-aortic CaPO_4_ application assessed by ultrasound and followed up for 28 days. Quantification graph showing mean and standard error of mean (SEM) analysed by Repeated measures two-way ANOVA and statistical significance shown as *P < 0.05. (**B**) Regional maximum diameters of *WT* and *KS-Tg* mice receiving peri-aortic application of CaPO_4_ determined by direct ex vivo morphometric measurements at the end of the 28 days study period. Ex vivo measurement of mean maximum aortic diameter showed that *KS-Tg* mice receiving CaPO_4_ resulted in significantly smaller IRA diameters when compared to the *WT* control mice. Quantification graph showing median and interquartile ranges (whiskers) analysed by Mann Whitney *U* test and statistical significance shown as **P = 0.01. (**C, D**) Effect of rhKAL on AngII-induced aortic dilatation in *ApoE*^−/−^ mice. (**C**) Shown are maximum external SRA diameter of *ApoE*^−/−^ administered with VC (n = 20) or rhKAL (n = 20), both subjected to 28 days of AngII-infusion. *P < 0.05. (**D**) Regional maximum diameters of *ApoE*^*−−-*^ mice administered with either VC or rhKAL determined by direct ex vivo morphometric measurements at the end of 28 days of AngII infusion. Ex vivo measurement of aortic arch, thoracic, SRA and IRA regions showed that mice receiving rhKAL had a significantly smaller maximum diameter when compared to controls. Quantification graph showing median and interquartile ranges (whiskers) and statistical significance shown as *P < 0.05, **P < 0.01, ***P < 0.001. IRA, infrarenal aortic diameter; *KS-Tg*, kallistatin transgenic; rhKAL, recombinant human KAL; SRA, suprarenal aortic; Trx, thoracic; VC, vehicle control; *WT*, wild type; AngII, angiotensin II; *ApoE*^−/−^, apolipoprotein E deficient; CaPO_4_, calcium phosphate.

AAA was induced by application of CaPO_4_ to the IRA of the *KS-Tg* and *WT* mice and expansion of the IRA was assessed by ultrasound and morphometry. Ultrasound imaging suggested that prior to CaPO_4_ application the maximum diameter of the IRA was similar in *KS-Tg* and *WT* mice. Following application of CaPO_4_ the external diameter of the IRA increased significantly more in *WT* than in *KS-Tg* mice over 28 days (*p* = 0.047; Fig. 1A, Supplementary Fig. S3) when assessed by ultrasound.

**Figure 2:**
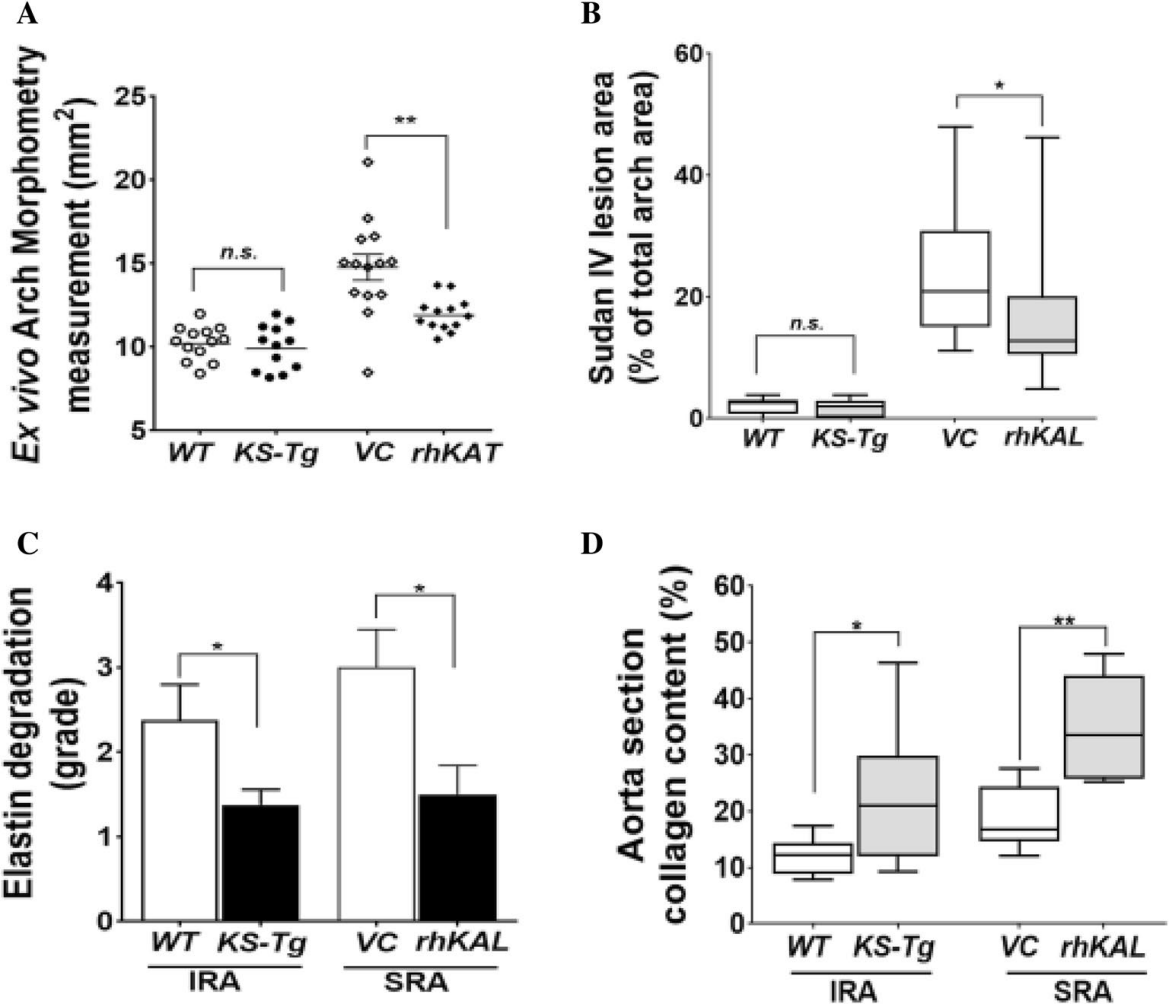
The effect of KAL in the aorta of CaPO_4_ induced and AngII-induced AAA mouse models. (**A**) Ex vivo morphometry measurement of ascending aortic aneurysm measured using an e*n face* method. (**B**) Percent atherosclerosis lesion area of thoracic aorta measured by *en face* Sudan IV staining method. (**C**) Quantification graph showing elastin filament degradation (n = 6 aorta/group). Aortic wall elastin filament degradation was graded based on the degree of breaks in elastin filaments (graded on a scale of 1–4) as described in the materials and methods. (**D**) Quantification of polarisation images for collagen content expressed as a percentage (%) of the total IRA and SRA section areas in the CaPO_4_ and AngII models, respectively (n = 6 aorta/group). Data shown as median and interquartile range and analysed by Mann–Whitney U test. Statistical significance shown as *P < 0.05*,* **P < 0.01. AngII, angiotensin II; n.s., not significant; rhKAL, recombinant human Kallistatin; *Kal-Tg*, kallistatin transgenic; SRA, suprarenal aortic diameter; IRA, infrarenal aortic diameter; VC, vehicle control; *WT*, wild type; *ApoE*^−/−^, apolipoprotein E deficient; CaPO_4_, calcium phosphate.

At the end of the experiments, aortas were harvested and examined ex vivo. Visible IRA dilation was noted in 6 out of 13 *WT* mice and in one of the 12 *KS-Tg* mice (Supplementary Fig. S4). Morphometric assessment showed that the *WT* mice had a mean (± SEM) maximum exterior IRA diameter of 1.29 ± 0.15 mm compared to 0.79 ± 0.03 mm for *KS-Tg* mice (*p* = 0.010; Fig. 1B). *En face* measurement of intimal surface area of the ascending aorta was used as an index to determine ascending aortic dilation. No difference between genotypes was observed (*p* = 0.586; Fig. 2A; Supplementary Fig S5.A).

### Administration of recombinant human KAL inhibited AngII-induced AAA in ApoE^−/−^ mice

AngII-infusion induced fatal aortic rupture in 13 of the 40 mice (33%) within 14 days. Administration of rhKAL did not influence survival free from aortic rupture (Supplementary Fig. S6). Ultrasound imaging demonstrated that in surviving mice AngII-induced increase in SRA diameter was significantly inhibited by rhKAL administration (*p* = 0.030; Fig. 1C).

Ex vivo external diameter measurement of harvested aortas from all surviving mice demonstrated that mice receiving rhKAL had significantly smaller maximum external SRA diameter. The mean (SEM) external SRA diameter of the control group (2.48 ± 0.18 mm) was significantly greater than that of the mice receiving rhKAL (1.77 ± 0.20 mm, *p* = 0.009; Fig. 1D; Supplementary Fig. S7). A smaller maximum external diameter was also observed within the aortic arch (*p* < 0.001), thoracic aorta (*p* < 0.001) and IRA (*p* = 0.017) in the AngII-infused *ApoE*^−/−^ mice receiving rhKAL compared to the control group (Fig. 1D). Subsequent assessment of *en face* measurement of intimal surface area of the ascending aorta showed a corresponding significantly smaller mean intimal ascending aortic area in AngII-infused *ApoE*^−/−^ mice receiving rhKAL compared to the control group (*p* = 0.003; Fig. 2A; Supplementary Fig S5.B).

### KAL limited atherosclerosis in the AngII-infused not the CaPO4 model

The systolic blood pressure of *KS-Tg* and *WT* mice was not significantly different (Supplementary Fig. S8.A). Body weight of *KS-Tg* mice was greater than *WT* mice throughout the experiment (Supplementary Fig. S9.A). rhKAL administration did not significantly affect systolic blood pressure or body weight (Supplementary Fig. S8.B and Fig. S9.B). No difference was observed between the *WT* and the *KS-Tg* genotypes in Sudan IV *enface* staining (Fig. 2B). The rhKAL administered group had a significantly lower staining area compared to the VC group, with a median staining area of 20.90% (1QR 15.05–30.92) versus 12.78% (IQR 10.53–20.13, *p* = 0.016; Fig. 2B).

### Transgenic overexpression of KAL reduced plasma concentrations of inflammatory cytokines in the CaPO_4_ AAA model

Out of the six cytokines assessed, significantly lower concentrations of TNF-α, INF-γ, IL-10 and IL-12 where found in the plasma of *KS-Tg* compared to the *WT* mice at the end of the experiment (Table 3). In contrast, the administration of rhKAL in the AngII-*ApoE*^−/−^ model did not significantly affect plasma concentrations of these cytokines (Table 3).

**Table 3:** Concentrations of cytokines (pg/ml) in the plasma of AAA animal models at the end of the 28 days experiment period.

Samples	CaPO_4_ AAA model			AngII AAA model		
	WT (n = 8)	KS-Tg (n = 8)	*P* value	VC (n = 8)	rhKAL (n = 8)	*P* value
TNFα	16.54 (6.67–90.01)	4.13 (2.11–6.57)	0.005	3.97 (3.63–7.68)	5.13 (4.05–5.97)	0.688
IL-12p70	75.25 (10.62–285.00)	8.00 (0–13.82)	0.004	7.86 (1.98–18.24)	9.36 (6.22–20.02)	0.548
IFN-γ	3.25 (0.96–12.64)	0.73 (0.32–1.08)	0.007	0.78 (0.47–1.31)	0.73 (0.62–1.17)	0.362
IL-10	41.33 (0–207.20)	*ND*	0.015	*ND*	*ND*	
MCP-1	59.70 (22.39–129.80)	20.85 (0–76.17)	0.207	35.71 (34.18–221.20)	31.65 (5.34–255.80)	0.571
IL-6	7.51 (3.62–47.81)	6.22 (2.23–13.30)	0.481	5.42 (3.70–8.23)	5.66 (3.12–14.84 )	> 0.999

Data presented as median (interquartile range, IQR) and statistical analysis performed using Mann Whitney U test. P value of < 0.05 was considered significant.

AAA, Abdominal aortic aneurysm*;* AngII, angiotensin II; CaPO_4_, calcium phosphate; IFN-γ, Interferon gamma; IL, Interleukin; *KS-Tg*, kallistatin transgenic; MCP-1, Monocyte Chemoattractant Protein-1; ND, not detected; rhKAL, recombinant human Kallistatin; TNF, Tumour Necrosis Factor; VC, vehicle control; *WT*, wild type.

### KAL reduced aortic wall remodelling in the mouse models

The degree of elastin filament disruption was significantly greater within the IRA or SRA from the *WT* compared with the *KS-Tg* or mice receiving rhKal compared to controls (Fig. 2C, Supplementary Fig. S10). The collagen content was assessed by polarisation microscopy revealing significantly higher collagen content in the IRA of *KS-Tg* (median staining area 20.90%, IQR 12.04–29.86) compared to *WT* mice (median staining area 12.24%, IQR 8.78–14.29; *p* = 0.040; Fig. 2D, Supplementary Fig. S10). Similarly, a higher collagen birefringence was noted in the SRA of mice receiving rhKAL (median staining area 33.52%, IQR 25.80–44.10) compared to the control group (median staining area 16.82%, IQR 14.64–24.36; *p* = 0.009; Fig. 2D).

### KAL attenuated oxidative stress and VSMC apoptosis

**Figure 3:**
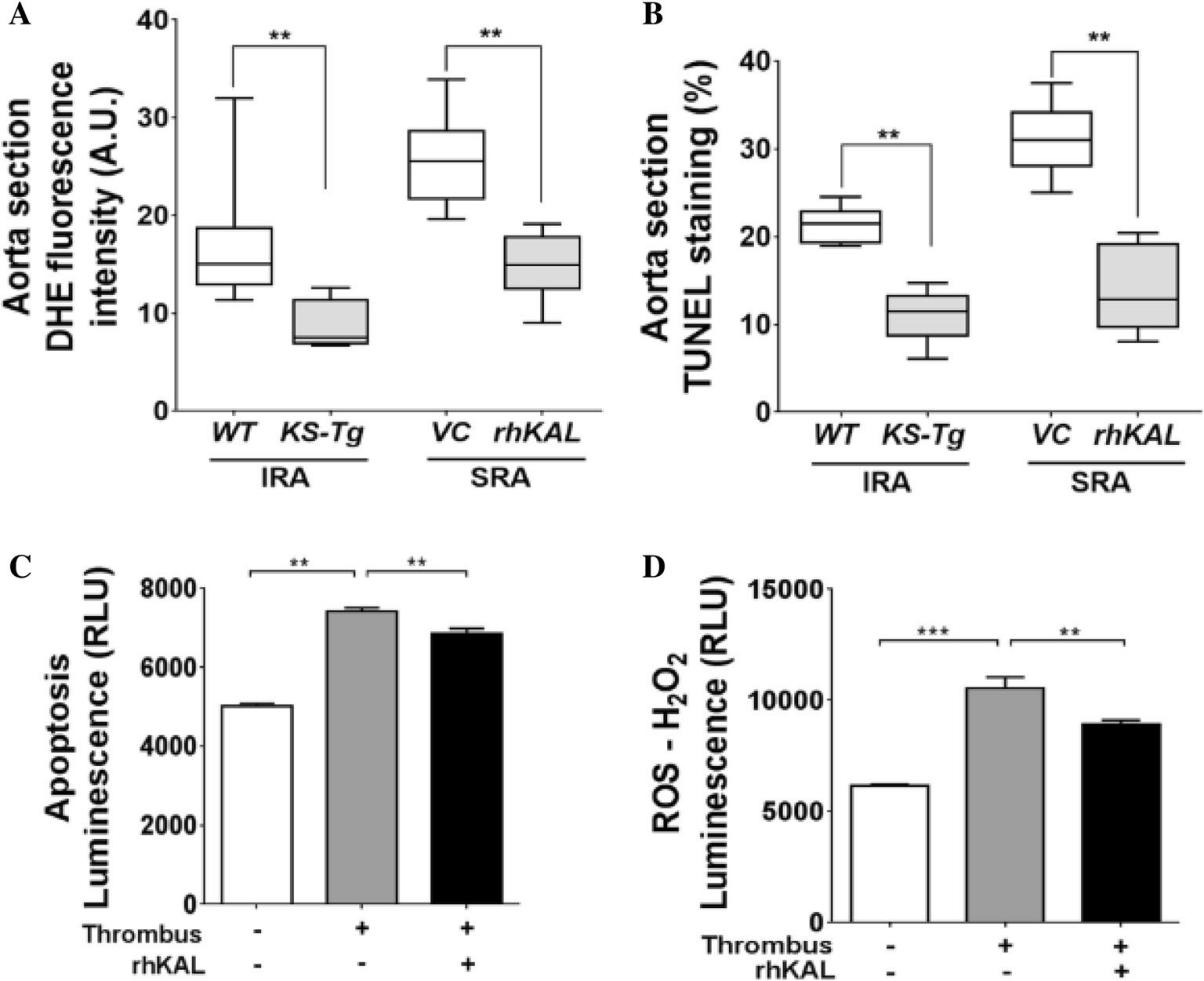
The effect of KAL on oxidative stress and apoptosis in CaPO_4_ induced and AngII-induced AAA mouse models and in vitro. (**A**) Quantitative data showing mean fluorescence was quantified and expressed as DHE staining intensity (%). Data expressed as median and interquartile range with maximum and minimum data points (whiskers) for positive staining area relative to total specimen area (%); *P < 0.05*,* **P < 0.01 by Mann–Whitney *U* test (n = 6 aorta/group). (**B**) Quantitative data showing TUNEL staining, shown is data expressed as median and interquartile range with maximum and minimum data points for positive staining area relative to total specimen area (%); **P < 0.01 by Mann–Whitney *U* test (n = 6 aorta/group). (**C, D**) VSMCs were plated at 1 × 10^6^ cells/ml in 500 µl DMEM + 5% FBS and allowed to adhere overnight. Exposure of VSMCs to AAA thrombus-derived conditioned medium for 24 h (n = 6/group) promoted both apoptosis (**C**) and upregulation of ROS activity (**D**) in these cells as assessed by mean relative luminescence unit (RLU)*.* Addition of 10 nM rhKAL to the conditioned media resulted in significant reduction in both apoptosis and ROS activity (**C**,**D**). All experiments were performed in triplicate (n = 6/group). Analysis performed by Kruskal Wallis test and post hoc analysis and statistical significance shown as *P < 0.05*;* **P < 0.01; ***P < 0.001*.* AngII, angiotensin II; DHE, dihydroethidium; *Kal-Tg*, kallistatin transgenic; rhKAL, recombinant human Kallistatin; SRA, suprarenal aortic diameter; IRA, infrarenal aortic diameter; ROS, reactive oxygen species; VC, vehicle control; *WT*, wild type.

Elevated oxidative stress was identified by the number of DHE-positive cells in the aortic wall. CaPO_4_ administration and AngII infusion led to marked DHE staining which was reduced by transgenic KAL [*WT* median DHE intensity, 15.04AU (IQR 12.85–18.82) versus *KS-Tg* median DHE intensity 7.55AU (IQR 6.77–11.49), *p* = 0.002; Supplementary Fig. S11] and administration of rhKAL [VC 25.58AU (IQR 21.62–28.77) versus rhKAL 14.97AU (IQR 12.36–17.89), *p* = 0.002; Fig. 3A].

Median (IQR) of TUNEL staining area was significantly higher in sections of the IRA of *WT* mice compared to *KS-Tg* mice (21.50%, IQR 19.16–23.05 vs. 11.46%, 8.61–13.41, *p* = 0.002; Fig. 3B; Supplementary Fig. S11). Similarly, median (IQR) TUNEL staining area was higher in the SRA sections from VC group (30.98%, IQR 27.87–34.30) compared to the rhKAL administered group (12.84%, 9.61–19.28, *p* = 0.007; Fig. 3B). The TUNEL positive apoptotic cells were mostly located in the medial region of IRA and SRA suggesting these were VSMCs. Hence, in vitro VSMC studies were conducted. Incubating VSMCs with human AAA-thrombus derived conditioned medium for 24 h promoted apoptosis in these cells as assessed by mean relative luminescence units (RLU, *p* = 0.001; Fig. 3C). Addition of 10 nM rhKAL to conditioned media-exposed VSMCs for 24 h resulted in a significant reduction in apoptosis (*p* = 0.009; Fig. 3C). In addition, exposure of VSMCs to AAA-thrombus derived conditioned medium for 24 h resulted in marked upregulation of ROS activity (*p* < 0.001; Fig. 3D), an effect that was significantly reduced in the presence of rhKAL (10 nM RLU, *p* = 0.001; Fig. 3D).

### KAL attenuated expression of ECM remodelling genes

QRT-PCR analysis was performed to assess the relative expression of *Mmp-9*, *Mmp-2*, *Opg, Opn* and *Vegf* in the IRA and SRA samples from both mouse studies. The relative expressions of *Mmp-9*, *Mmp-2* and *Opn* were significantly downregulated in the IRA samples of *KS-Tg* compared to *WT* mice (Table 4), however, remained comparable in AngII-infused *ApoE*^−/−^ mice receiving rhKAL or vehicle control (Table 4).

**Table 4:** Relative gene expressions in the aortas of *WT* and *KS-Tg* mice in the CaPO_4_ model and mice receiving vehicle control or rhKAL in the AngII-infused *ApoE*^*−−-*^ mouse model after 28 days.

Genes	Genotype	Median expression (IQR)	*P* value	Genotype	Median expression (IQR)	*P* value
Mmp-9	WT	2.23 (1.32–6.78)	0.016	VC	1.45 (1.27–2.03)	0.468
	KS-Tg	1.01 (0.37–2.01)		rhKAL	1.80 (0.83–3.30)	
Mmp-2	WT	1.00 (0.28–3.40)	0.035	VC	0.47 (0.26–0.96)	0.721
	KS-Tg	0.44 (0.16–0.90)		rhKAL	0.75 (0.28–1.11)	
Opn	WT	1.16 (0.67–3.09)	0.041	VC	0.37 (0.28–0.78)	0.232
	KS-Tg	0.56 (0.21–0.79)		rhKAL	0.28 (0.03–0.59)	
Opg	WT	2.56 (1.07–17.40)	0.114	VC	2.67 (1.64–6.24)	0.072
	KS-Tg	1.06 (0.50–1.97)		rhKAL	0.75 (0.40–3.86)	
Vegf	WT	1.50 (0.56–2.29)	0.104	VC	1.06 (0.34–1.70)	0.613
	***KS-Tg***	0.95 (0.38–1.17)		***rhKAL***	0.81 (0.43–2.53)	

CaPO_4_ induced AAA model was developed in *WT* and *KS-Tg* mice and received peri-aortic administration of CaPO4. AngII-induced AAA model received daily subcutaneous injection of VC or rhKAL for 28 days. Total RNA was isolated from randomly selected aorta tissues stored in RNAlater (n = 8/group) at the end of the study and gene expression was assessed by quantitative real time PCR. Data expressed as median (interquartile range, IQR). Results are compared by Mann Whitney u test and expressed as relative expression of gene compared to glyceraldehyde 3–phosphate dehydrogenase (*Gapdh*).

AAA, Abdominal aortic aneurysm*;* AngII, angiotensin II; CaPO_4_, calcium phosphate; *WT*, wild type; *KS-Tg*, kallistatin transgenic; rhKAL, recombinant human kallistatin; *Mmp*, matrix metallioproteinase; *Vegf*, vascular endothelial growth factor, *Opg*, osteoprotegerin, *Opn*, osteopontin; VC, vehicle control.

**Figure 4:**
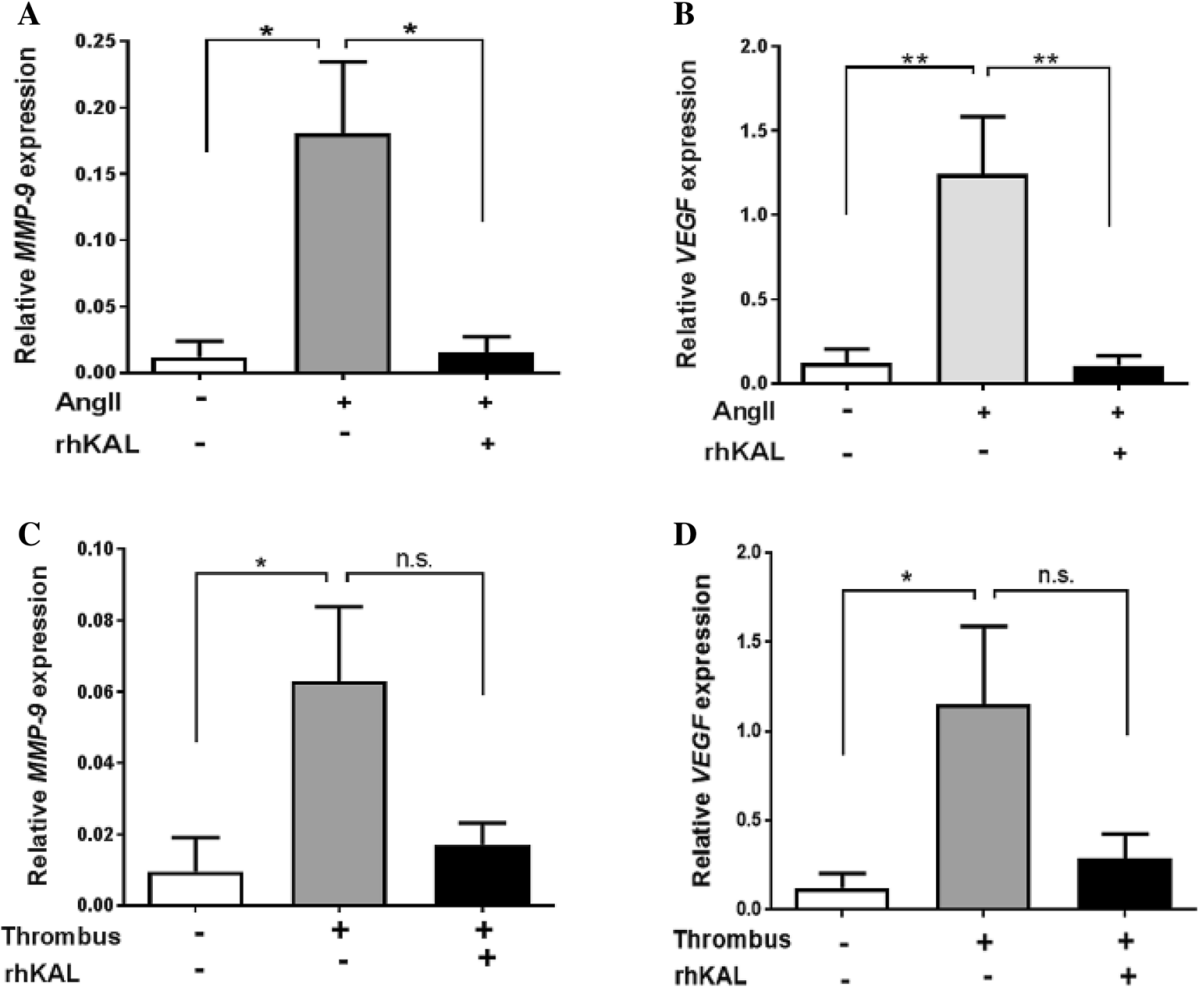
KAL attenuated the expression of extracellular matrix regulating genes in VSMCs. VSMCs were plated at 1 × 10^6^ cells/ml in 500 µl DMEM + 5% FBS and allowed to adhere overnight. Subsequently, the cells were incubated with 2 nm AngII or AAA thrombus-derived media for 24 h. At the end of the experiment, VSMCs were collected in the RNAlater and total RNA isolated to perform quantitative real time PCR (QRT–PCR). (**A**,**B**) Incubating VSMCs with 2 nm AngII resulted in significant upregulation of *MMP-9* and *VEGF* gene expressions. Co-incubation with 10 nM rhKAL for 24 h attenuated AngII-induced upregulation of *MMP-9* and *VEGF*. (**C**,**D**) VSMCs exposed to AAA thrombus-derived conditioned medium for 24 h promoted *MMP-9* and *VEGF* expression*.* Addition of 10 nM rhKAL to the conditioned media resulted in a significant reduction in both *MMP-9* and *VEGF* expressions. All experiments were performed in triplicate (n = 6/group). Analysis performed by Kruskal Wallis test and post-hoc analysis and statistical significance shown as *P < 0.05; **P < 0.01. AngII, angiotensin II; rhKAL, recombinant human kallistatin; *MMP*, Matrix metalloproteinase; *VEGF*, vascular endothelial growth factor; VSMC, vascular smooth muscle cell; n.s, non-significant.

VSMC dysfunction, in response to cytokines released from AAA-thrombus or to AngII, is thought to play an important role in dysregulated ECM remodelling associated with AAA^30,31^. Significant upregulation in *MMP-9* and *VEGF* expression in VSMCs cultured in the presence of AngII (2 nM) or with AAA-thrombus derived conditioned medium over 24 h was attenuated in the presence of rhKAL (10 nM; Fig. 4A–D).

### KAL upregulates SIRT1

**Figure 5:**
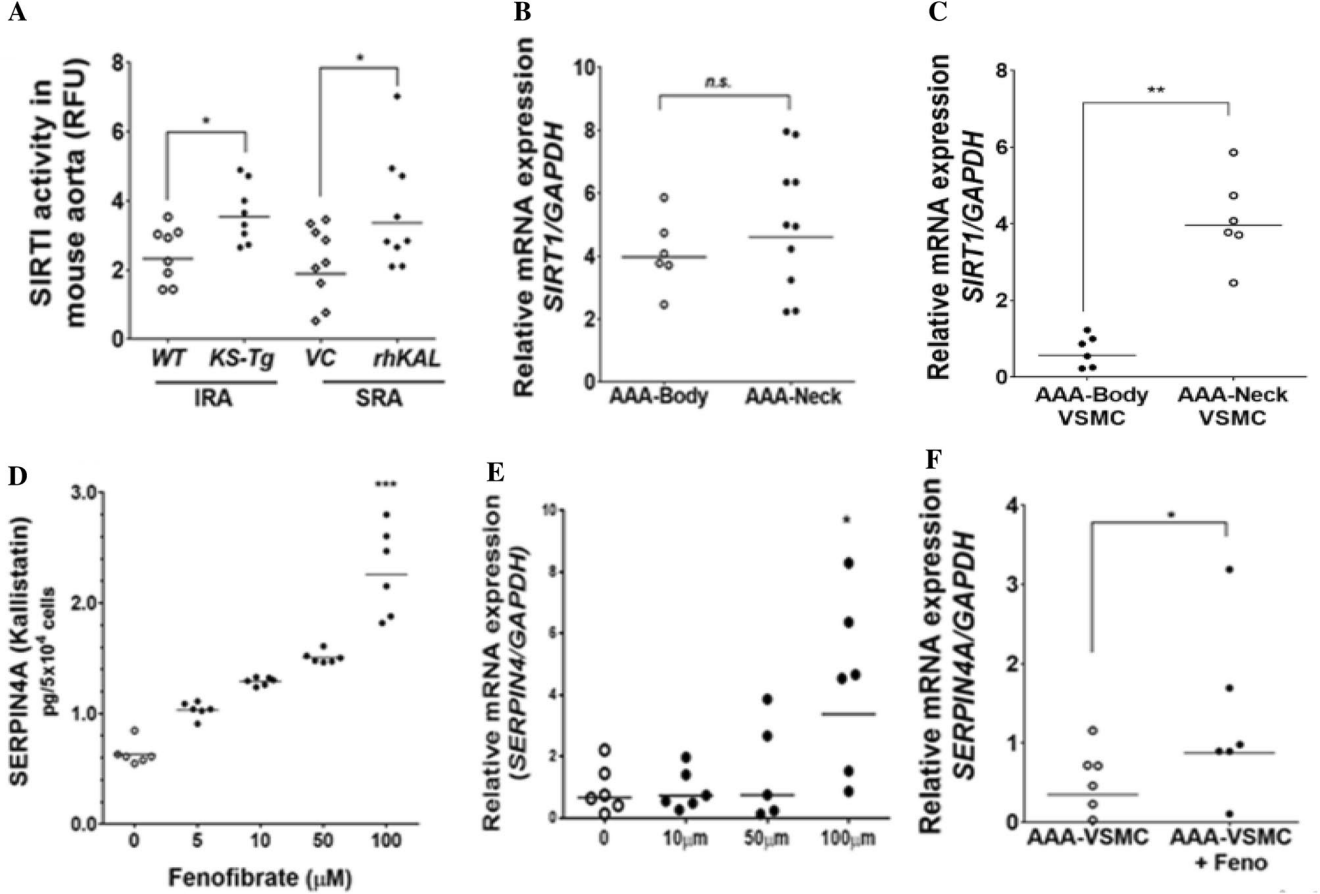
KAL upregulates SIRT1 activity and fenofibrate upregulates KAL expression. (**A**) SIRT1 activity was assessed in the nuclear extract from IRA and SRA segments. Compared to the respective controls, the nuclear protein extract obtained from the CaPO_4_ administered *KS-Tg* mice and rhKAL-administered AngII-induced *ApoE*^−/−^ mice showed increased SIRT1 activity (n = 8/group). (**B**) Relative *SIRTI* mRNA expression in AAA-neck (n = 6) and AAA-body (n = 12) tissues. Aortic tissues were collected in the RNAlater and total RNA isolated to perform quantitative real time PCR (QRT–PCR). (**C**) AAA-Body and AAA-neck tissues (n = 6) were used for isolating VSMCs using established protocol. AAA-VSMCs were collected in the RNA later and total RNA isolated to perform QRT-PCR to assess *SIRT1* mRNA expression. (**D**,**E**) Incubation of VSMCs with increasing concentration of fenofibrate upregulated KAL protein expression. Cells were plated at 0.5 × 10^6^ cells in 500 µl DMEM + 5% FBS and incubated with fenofibrate (0–100 µM) over 24 h after which protein and mRNA was assayed (n = 6/group). Incubation of VSMCs with increasing concentration of fenofibrate (0–100 µM) dose-dependently stimulated (**D**) SERPINA4 protein and (**E**) *SERPINA4* mRNA expression**.** Protein concentrations were assessed by Bradford method and SERPINA4 concentration levels were assessed in the cell culture supernatants by ELISA. QRT-PCR was performed on extracted total mRNA using *SERPIN4A* primers and normalised to *GAPDH* expression. (n = 6 replicates). (**F**) AAA-VSMCs (n = 6) were incubated with fenofibrate (100 µM) for 24 h. Total RNA was isolated from the cells *SERPINA4* mRNA expression was assessed by QRT-PCR. Analysis performed by Mann Whitney *U* or Kruskal Wallis tests and statistical significance shown as *P < 0.05; **P < 0.01; ***P < 0.001*. GAPDH,* glyceraldehyde 3 phosphate; IRA, infrarenal aorta; *KS-Tg*, kallistatin transgenic; rhKAL, recombinant human kallistatin; RFU, relative fluorescent unit; *SERPINA4,* serpin-A4*; SIRT1*, sirutuin-1; SRA, suprarenal aorta; VC*,* vehicle control; VSMC, vascular smooth muscle cell; *WT,* wild type.

KAL has been shown previously to reduce ROS production through the SIRT1 pathway^32^. Nuclear SIRT1 activity was assessed in IRA and SRA tissue from the mouse models. Nuclear protein extract obtained from *KS-Tg* mice and *ApoE*^−/−^ mice receiving rhKAL showed increased SIRT1 activity compared to controls (Fig. 5A). Expression of the *SIRT1* gene was assessed in AAA body and neck samples from patients and found to be similar (Fig. 5B). Subsequently, VSMCs isolated from paired AAA body and neck samples were assessed and found that AAA-VSMCs isolated from aneurysmal body had comparatively lower expression levels of *SIRT1* compared to VSMCs from relatively disease-free neck (Fig. 5C).

### Fenofibrate upregulates KAL in VSMCs

A previous study from our group found that fenofibrate effectively reduced AAA in the AngII model^26^. Fenofibrate has also been shown to ameliorate anti-oxidative effect by regulating the expression of oxidant and anti-oxidant enzymes^33^. Since SIRT1 expression was downregulated in AAA-derived VSMCs and it was previously reported that fenofibrate increases cardiac expression of SIRT1^34^, the effect of fenofibrate was examined on VSMCs. VSMCs were incubated with increasing concentrations of fenofibrate and the KAL protein concentration and *SERPINA4* mRNA in cell culture supernatant and VSMCs were measured. Fenofibrate (0–100 µM) dose-dependently increased supernatant concentrations of KAL protein (Fig. 5D) and stimulated *SERPINA4* expression compared to controls at the maximum dose of 100 µM (Fig. 5E). Subsequently, AAA-VSMCs were incubated with fenofibrate which significantly upregulated *SERPINA4* expression (Fig. 5F).

## Discussion

The main findings of this study were that serum KAL was negatively associated with AAA diagnosis and growth, and that within mouse models upregulating KAL reduced the size of AAAs. The findings suggest that KAL plays a protective role against AAA. A previous proteomic study identified KAL as one of the five proteins found to be differentially up-regulated in AAA patients^35^. This previous study included only a small number of hospital recruited patients under investigation for coronary artery disease (17 AAA patients and 17 controls). A more recent study also reported upregulation of KAL in AAA human samples when assessed by Western blotting but reported downregulation of KAL when analysed by real time PCR (36 AAA patients and 12 controls)^36^. Neither study analysed aortic cells. In contrast, in the current study we investigated VSMCs isolated from AAA patients and blood from approximately 1000 men aged over 65 years recruited from the community and screened for AAA. We found a negative association between serum KAL and AAA diagnosis and growth. The AAAs identified in this cohort were mostly small and therefore the findings are likely representative of the association of KAL with early stage AAA.

Inflammation is thought to play an important role in human AAA pathogenesis and several studies suggest that KAL limits inflammation^10,37^. Transgenic overexpression of KAL in mice resulted in significant reduction in the plasma levels of a number of cytokines including TNF-α, IFN-γ, IL-10 and IL-12 after AAA induction. TNF-α is considered a key stimulant of pro-inflammatory responses by promoting cytokine release, oxidative stress and angiogenesis^10^. IFN-γ is a cytokine that stimulates T cells and macrophages based immune responses^38^. IFN-γ concentrations have been reported to be increased in human AAA^39^. These effects may have been important in the relative AAA resistance of the *KS-Tg* mice but was not found in the mice receiving rhKAL and therefore other mechanisms were examined.

Elastin fragmentation, collagen destruction and VSMC apoptosis are important features of human AAA and have been implicated in AAA development within the CaPO_4_ and AngII models^21^. The histopathological assessment in the current study suggested that transgenic overexpression of KAL and administration of rhKAL limited elastin degradation and attenuated fibrosis. These findings are in line with a number of previous studies that suggested that KAL upregulation limits ECM remodelling^9,40–43^. Matrix regulating proteins such as MMPs, OPN, OPG and VEGF have been previously implicated in experimental and human AAA^44–47^. In the *KS-Tg* mice, but not mice receiving rhKAL, *Mmp-9*, *Mmp-2* and *Opn* expressions were downregulated. The disparate effects of the two methods of upregulating KAL is likely explained by the less powerful increase in KAL achieved by intermittent i.p. injection as compared to transgenic upregulation. Also the findings were likely contributed to by the different models used. The AngII infused AAA model has greater ECM remodelling and inflammation than the CaPO_4_ model which may have overwhelmed the effects of rhKAL.

Apoptosis of VSMCs is known to be important in experimental and human AAA^48,49^. In the current study, KAL upregulation limited DHE and TUNEL staining within the aorta in both AAA models. Furthermore, administration of rhKAL inhibited apoptosis of healthy VSMCs in vitro. Incubation of VSMC with rhKAL also inhibited ROS generation in these cells stimulated by exposure to human AAA-thrombus conditioned media or AngII. Taken together, the findings from the experimental mouse models and in vitro studies suggest that KAL inhibited AAA by blocking inflammation, ROS generation and VSMC apoptosis.

SIRT1 expression in VSMC is required to maintain the structural integrity of the aortic wall in response to oxidant and inflammatory stimuli^50^ and recent studies suggest that VSMC SIRT1 plays a protective role in the AngII-infused AAA model^51^. SIRT1 has been reported to protect against aortic rupture in response to AngII^52,53^. VSMC-specific knockout of *Sirt1* has been reported to accelerate AngII-induced formation and rupture, whereas VSMC-specific overexpression of *Sirt1* suppressed AngII-induced AAA formation and progression in *ApoE*^−/−^ mice. The current study suggests that KAL upregulation increased SIRT1 activity, which likely contributed to the reduced AAA severity in these mice. *SIRT1* expression in tissues obtained from human AAA-body and AAA-neck was similar. However, when a pure population of VSMCs isolated from the tissues were assessed, the *SIRT1* expression was shown to be significantly downregulated in AAA-body VSMCs compared to the VSMCs from the relatively normal region of the AAA-neck.

Fenofibrate is a peroxisome-proliferator-activated receptor-α agonist, which is used clinically to lower triglyceride levels. Previous mouse model studies suggest that fenofibrate inhibits AAA development^26,54^. The Fenofibrate in the Management of Abdominal Aortic Aneurysm (FAME)-2 trial showed that 24 weeks of fenofibrate therapy did not significantly influence the circulating concentrations of AAA-associated proteins including osteopontin and KAL despite reducing serum triglyceride^55^. However, the study was underpowered to examine the relation between fenofibrate and AAA growth. A further ongoing trial (FAME-1) is examining the effect of fenofibrate on the aortic wall. In the current study, fenofibrate upregulated KAL expression in VSMCs in vitro in a dose dependent manner, and a similar effect was observed in AAA-VSMCs, which could provide an approach by which fenofibrate might act to inhibit AAA progression.

The strengths of this study include the assessment of circulating KAL in a large number of men and the further examination of these findings in two rodent AAA models and in vitro studies. Some limitations of this study are acknowledged. First, since KAL has pleotropic functions, it is not possible to exclude the possibility that it limits AAA development by mechanisms not examined in this study. Further studies are needed to substantiate the findings in other populations, including women, and further experimental models. Secondly, systemic rhKAL administration was not effective in limiting inflammation unlike transgenic upregulation. This could be because rhKAL administration may not be as potent as the effect of transgenic overexpression of KAL although this was not specifically confirmed. The most appropriate way to deliver rhKAL to limit AAA needs to be further investigated.

In conclusion, this study suggests a protective role of KAL in AAA pathogenesis through limiting inflammation, ECM remodelling, ROS and VSMC apoptosis. The findings suggest that upregulating KAL maybe a therapeutic target to limit AAA development and progression.

## Supplementary Information

Supplementary Information.

## References

[1] Sampson UK (2014). Global and regional burden of aortic dissection and aneurysms: mortality trends in 21 world regions, 1990 to 2010. Glob. Heart.

[2] Chaikof EL (2018). The Society for Vascular Surgery practice guidelines on the care of patients with an abdominal aortic aneurysm. J. Vasc. Surg..

[3] Benson RA (2018). Ultrasound screening for abdominal aortic aneurysm: Current practice, challenges and controversies. Br. J. Radiol..

[4] Cao P (2011). Comparison of surveillance versus aortic endografting for small aneurysm repair (CAESAR): Results from a randomised trial. Eur. J. Vasc. Endovasc. Surg..

[5] United Kingdom Small Aneurysm Trial (2002). Long-term outcomes of immediate repair compared with surveillance of small abdominal aortic aneurysms. N. Engl. J. Med..

[6] Golledge J, Norman PE (2011). Current status of medical management for abdominal aortic aneurysm. Atherosclerosis.

[7] Golledge J (2019). Lack of an effective drug therapy for abdominal aortic aneurysm. J. Intern. Med..

[8] Golledge J (2019). Abdominal aortic aneurysm: Update on pathogenesis and medical treatments. Nat. Rev. Cardiol..

[9] Chao J (2001). Novel roles of kallistatin, a specific tissue kallikrein inhibitor, in vascular remodeling. Biol. Chem..

[10] Li J, Krishna SM, Golledge J (2016). The potential role of kallistatin in the development of abdominal aortic aneurysm. Int. J. Mol. Sci..

[11] Krishna SM (2016). Wnt signaling pathway inhibitor sclerostin inhibits Angiotensin II-induced aortic aneurysm and atherosclerosis. Arterioscler. Thromb. Vasc. Biol..

[12] Moran CS (2017). Resveratrol inhibits growth of experimental abdominal aortic aneurysm associated with upregulation of angiotensin-converting enzyme 2. Arterioscler. Thromb. Vasc. Biol..

[13] Liu X (2013). Antiangiogenic and antineuroinflammatory effects of kallistatin through interactions with the canonical Wnt pathway. Diabetes.

[14] Guo Y (2015). Kallistatin inhibits TGF-beta-induced endothelial-mesenchymal transition by differential regulation of microRNA-21 and eNOS expression. Exp. Cell Res..

[15] Yao Y (2018). Reduced plasma kallistatin is associated with the severity of coronary artery disease, and kallistatin treatment attenuates atherosclerotic plaque formation in mice. J. Am. Heart Assoc..

[16] Norman PE (2009). Cohort profile: The health in men study (HIMS). Int. J. Epidemiol..

[17] Biros E (2014). Differential gene expression in the proximal neck of human abdominal aortic aneurysm. Atherosclerosis.

[18] Golledge J (2007). Obesity, adipokines, and abdominal aortic aneurysm: Health in Men study. Circulation.

[19] Norman P (2004). C-reactive protein levels and the expansion of screen-detected abdominal aortic aneurysms in men. Circulation.

[20] Golledge J (2010). Association between serum lipoproteins and abdominal aortic aneurysm. Am. J. Cardiol..

[21] Wang Y (2014). Influence of apolipoprotein E, age and aortic site on calcium phosphate induced abdominal aortic aneurysm in mice. Atherosclerosis.

[22] Yamanouchi D (2012). Accelerated aneurysmal dilation associated with apoptosis and inflammation in a newly developed calcium phosphate rodent abdominal aortic aneurysm model. J. Vasc. Surg..

[23] Biros E (2015). Differential gene expression in human abdominal aortic aneurysm and aortic occlusive disease. Oncotarget.

[24] Rush C (2009). Whole genome expression analysis within the angiotensin II-apolipoprotein E deficient mouse model of abdominal aortic aneurysm. BMC Genomics.

[25] Chen X (2013). Amlodipine reduces AngII-induced aortic aneurysms and atherosclerosis in hypercholesterolemic mice. PLoS One.

[26] Krishna SM (2012). Fenofibrate increases high-density lipoprotein and sphingosine 1 phosphate concentrations limiting abdominal aortic aneurysm progression in a mouse model. Am. J. Pathol..

[27] Wang W (2019). Apelin protects against abdominal aortic aneurysm and the therapeutic role of neutral endopeptidase resistant apelin analogs. Proc. Natl. Acad. Sci. USA.

[28] Golledge J (2011). Evaluation of the diagnostic and prognostic value of plasma D-dimer for abdominal aortic aneurysm. Eur. Heart J..

[29] Golledge J (2008). Reduced expansion rate of abdominal aortic aneurysms in patients with diabetes may be related to aberrant monocyte–matrix interactions. Eur. Heart J..

[30] Sagan A (2012). Local inflammation is associated with aortic thrombus formation in abdominal aortic aneurysms. Relationship to clinical risk factors. Thromb. Haemost..

[31] Brasier AR, Recinos A, Eledrisi MS (2002). Vascular inflammation and the renin-angiotensin system. Arterioscler. Thromb. Vasc. Biol..

[32] Yiu WH (2016). Kallistatin protects against diabetic nephropathy in db/db mice by suppressing AGE-RAGE-induced oxidative stress. Kidney Int..

[33] Wang Y (2019). Fenofibrate improved interstitial fibrosis of renal allograft through inhibited epithelial-mesenchymal transition induced by oxidative stress. Oxid. Med. Cell Longev.

[34] Zhang J (2016). Fenofibrate increases cardiac autophagy via FGF21/SIRT1 and prevents fibrosis and inflammation in the hearts of Type 1 diabetic mice. Clin. Sci. (Lond).

[35] Acosta-Martin AE (2011). Quantitative mass spectrometry analysis using PAcIFIC for the identification of plasma diagnostic biomarkers for abdominal aortic aneurysm. PLoS One.

[36] He Y (2020). Kallistatin correlates with inflammation in abdominal aortic aneurysm and suppresses its formation in mice. Cardiovas. Diagnosis Therapy.

[37] Yin H (2010). Kallistatin inhibits vascular inflammation by antagonizing tumor necrosis factor-alpha-induced nuclear factor kappaB activation. Hypertension.

[38] Schoenborn JR, Wilson CB (2007). Regulation of interferon-gamma during innate and adaptive immune responses. Adv. Immunol..

[39] Szekanecz Z (1994). Human atherosclerotic abdominal aortic aneurysms produce interleukin (IL)-6 and interferon-gamma but not IL-2 and IL-4: The possible role for IL-6 and interferon-gamma in vascular inflammation. Agents Actions.

[40] Huang X (2014). Protection effect of kallistatin on carbon tetrachloride-induced liver fibrosis in rats via antioxidative stress. PLoS One.

[41] Diao Y (2011). Protection of the liver against CCl4-induced injury by intramuscular electrotransfer of a kallistatin-encoding plasmid. World J. Gastroenterol..

[42] Huang X (2017). Kallistatin protects against bleomycin-induced idiopathic pulmonary fibrosis by inhibiting angiogenesis and inflammation. Am. J. Transl. Res..

[43] Shen B (2008). Salutary effect of kallistatin in salt-induced renal injury, inflammation, and fibrosis via antioxidative stress. Hypertension.

[44] Koole D (2012). Osteoprotegerin is associated with aneurysm diameter and proteolysis in abdominal aortic aneurysm disease. Arterioscler. Thromb. Vasc. Biol..

[45] Moran CS (2014). Osteoprotegerin deficiency limits angiotensin II-induced aortic dilatation and rupture in the apolipoprotein E-knockout mouse. Arterioscler. Thromb. Vasc. Biol..

[46] Moran CS (2016). Modulation of Kinin B2 receptor signaling controls aortic dilatation and rupture in the angiotensin II-infused apolipoprotein E-deficient mouse. Arterioscler. Thromb. Vasc. Biol..

[47] Filis K (2014). Osteopontin and osteoprotegerin as potential biomarkers in abdominal aortic aneurysm before and after treatment. Int. Sch. Res. Notices.

[48] Henderson EL (1999). Death of smooth muscle cells and expression of mediators of apoptosis by T lymphocytes in human abdominal aortic aneurysms. Circulation.

[49] Metghalchi S (2018). Indoleamine 2 3-dioxygenase knockout limits angiotensin II-induced aneurysm in low density lipoprotein receptor-deficient mice fed with high fat diet. PLoS One.

[50] Zhang S (2018). SIRT6 protects against hepatic ischemia/reperfusion injury by inhibiting apoptosis and autophagy related cell death. Free Radic. Biol. Med..

[51] Liu Y (2016). Calorie restriction protects against experimental abdominal aortic aneurysms in mice. J. Exp. Med..

[52] Chen HZ (2016). Age-associated sirtuin 1 reduction in vascular smooth muscle links vascular senescence and inflammation to abdominal aortic aneurysm. Circ. Res..

[53] Fry JL (2015). Vascular smooth muscle sirtuin-1 protects against aortic dissection during angiotensin II-induced hypertension. J. Am Heart Assoc..

[54] Golledge J (2010). Peroxisome proliferator-activated receptor ligands reduce aortic dilatation in a mouse model of aortic aneurysm. Atherosclerosis.

[55] Pinchbeck JL (2018). Randomized placebo-controlled trial assessing the effect of 24-week fenofibrate therapy on circulating markers of abdominal aortic aneurysm: Outcomes from the FAME-2 Trial. J. Am. Heart Assoc..

