# Supplementary material for: Kallistatin limits abdominal aortic aneurysm by attenuating generation of reactive oxygen species and apoptosis
Source: Sci Rep. 2021 Aug 31;11:17451. doi: 10.1038/s41598-021-97042-8 (PMC8408144; doi:10.1038/s41598-021-97042-8)
Supplement: Supplementary file 1 — Supplementary Information. [file 41598_2021_97042_MOESM1_ESM.pdf]

SUPPLEMENTAL MATERIAL

Figures and Tables

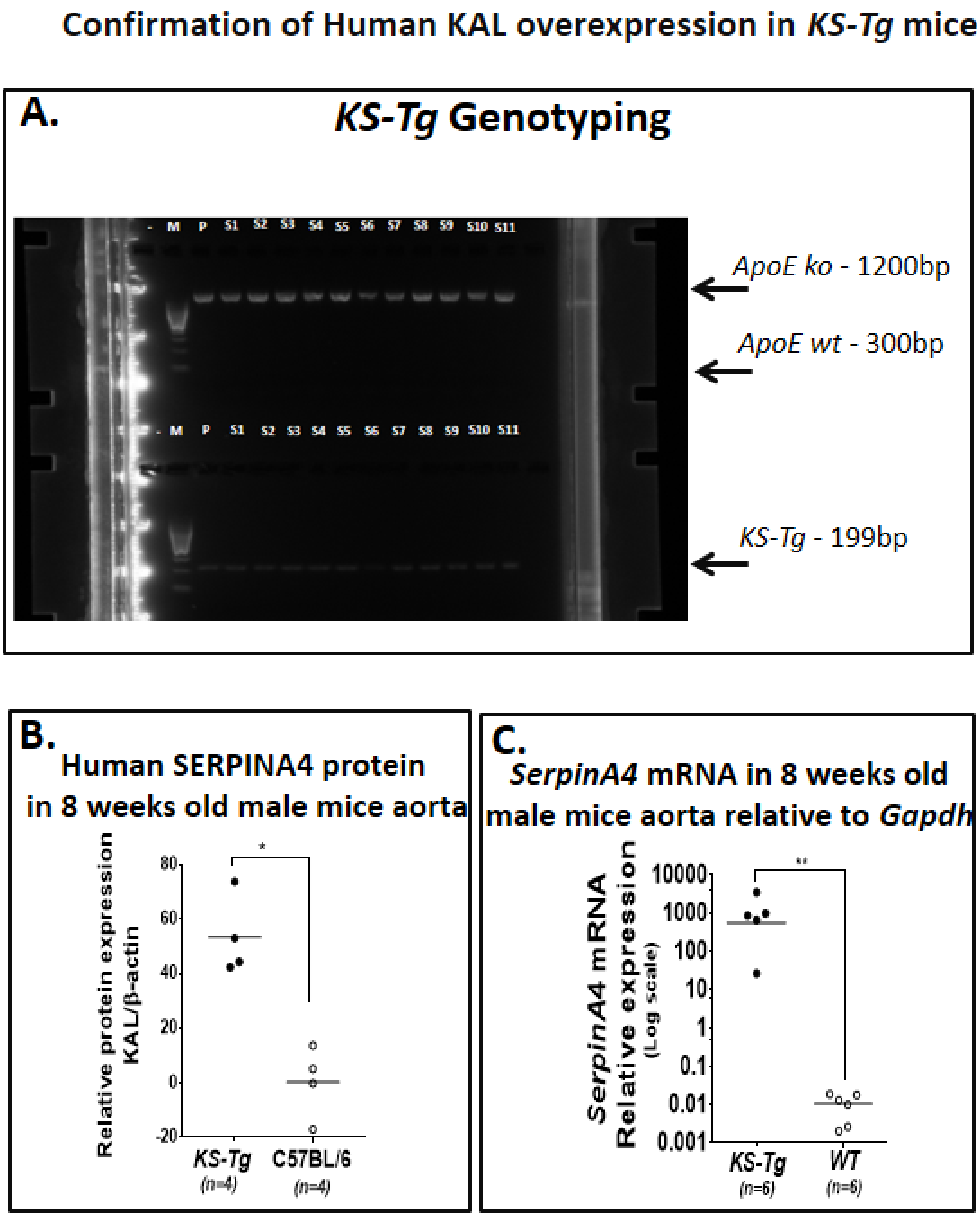

Figure S1. Confirmation of Human KAL expression in KS-Tg mice. A. Genotype assessed by gel electrophoresis. B. Graph showing quantitative Western blotting of the presence of human SERPINA4 protein (product of human kallistatin gene SERPINA4) in the aorta of KS-Tg genotype. C. Quantitative RT-PCR assessment of SerpinA4m RNA expression in the KS-Tg mice relative to Gapdh. ***Abbreviations:*** "-", negative, M, marker/100 bp ladder; P, positive control; S1 - S6, DNA samples. WT, wild type; KS-Tg, Kallistatin transgenic; ApoE ko, Apolipoprotein E knock out. *P<0.05; **P<0.01.

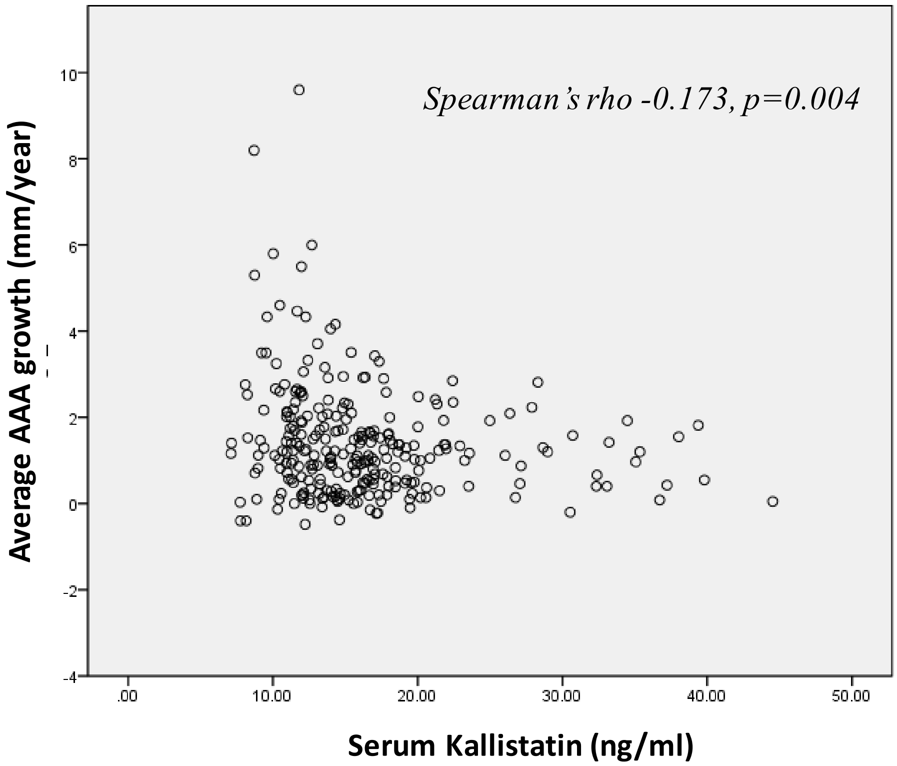

Figure S2. **AAA growth is negatively correlated with serum KAL concentration.** Graph showing inverse correlation between average AAA growth (mm/year) and serum KAL levels in AAA patients (n=272).

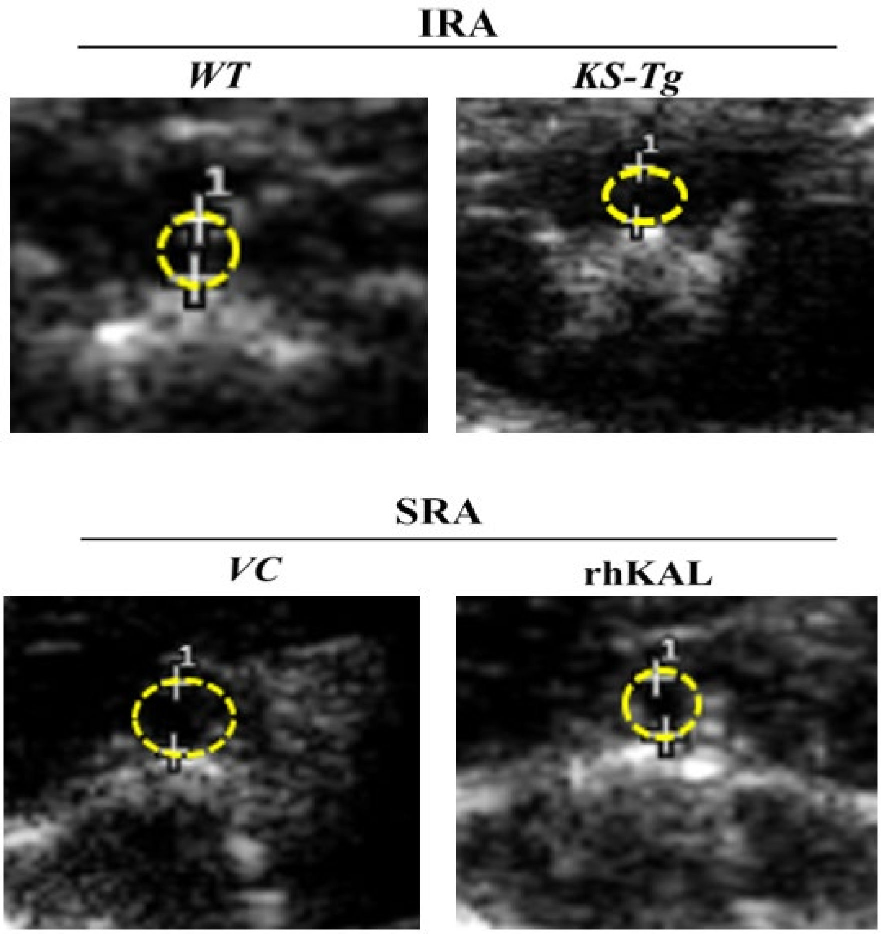

Figure S3: Representative ultrasound images. (A) Infra renal aorta (IRA) measurements in $CaPO_4$-AAA model, and (B) Suprarenal aorta (SRA) measurement of AngII-AAA model. The yellow dotted circle represent the aorta lumen, and '+' denotes the callipers used for measurements by the software.

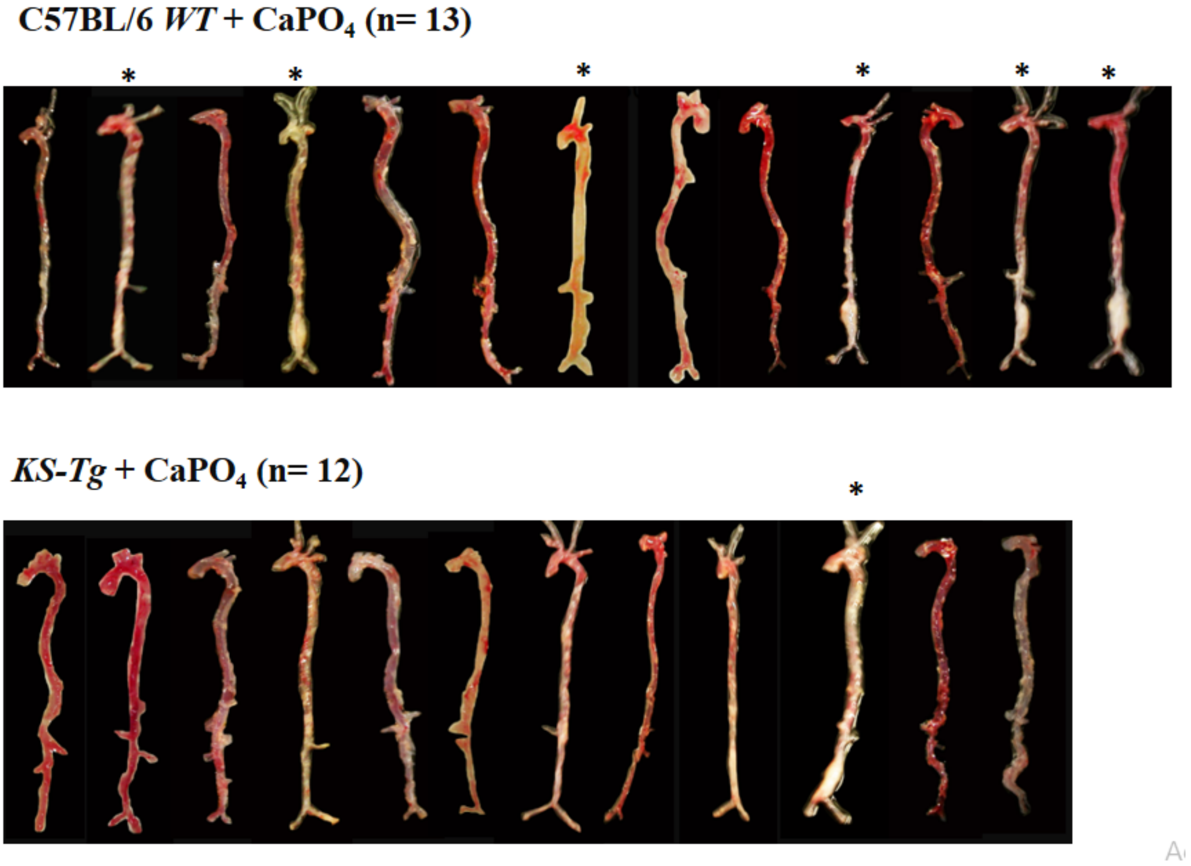

Figure S4: Effect of transgenic overexpression of human Kallistatin (KAL) gene on aortic response to calcium phosphate ($CaPO_4$). Gross morphology of infrarenal aortas (IRA) harvested from wild type *C57BL/6* and mice with transgenic introduction of KAL (*KS-Tg*) mice after 28 days, in which aneurysms were induced by perivascular application of 0.5 M calcium chloride ($CaCl_2$) followed by phosphate buffered saline. '*' denotes the IRAs with visible dilations.

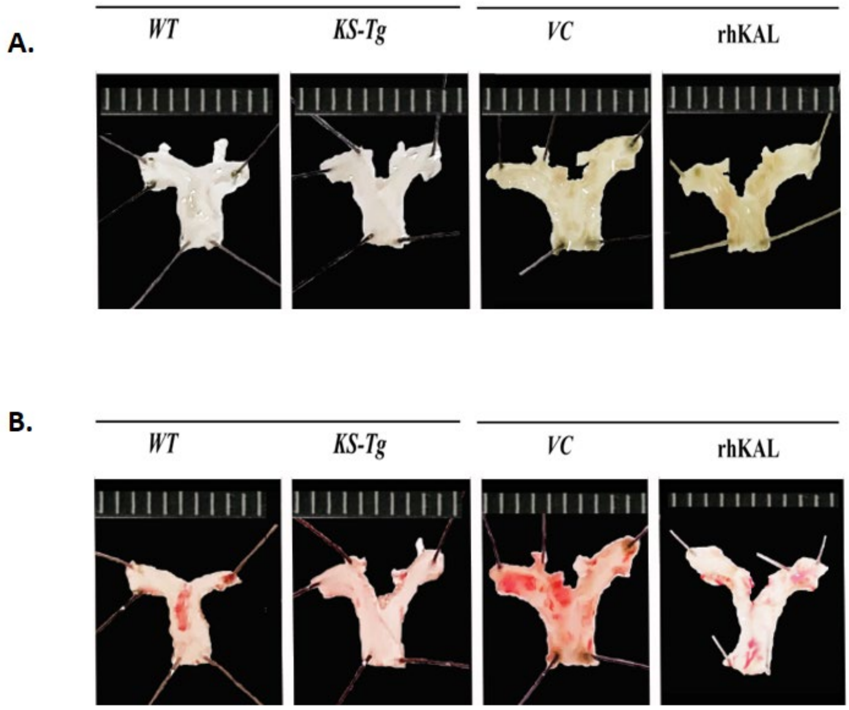

Figure S5: Representative photographs of excised aortic arch segments. A. Representative images of aortic arches processed for measurement of intimal area**.** B. Images of *en face* Sudan IV staining of aortic arch segments.

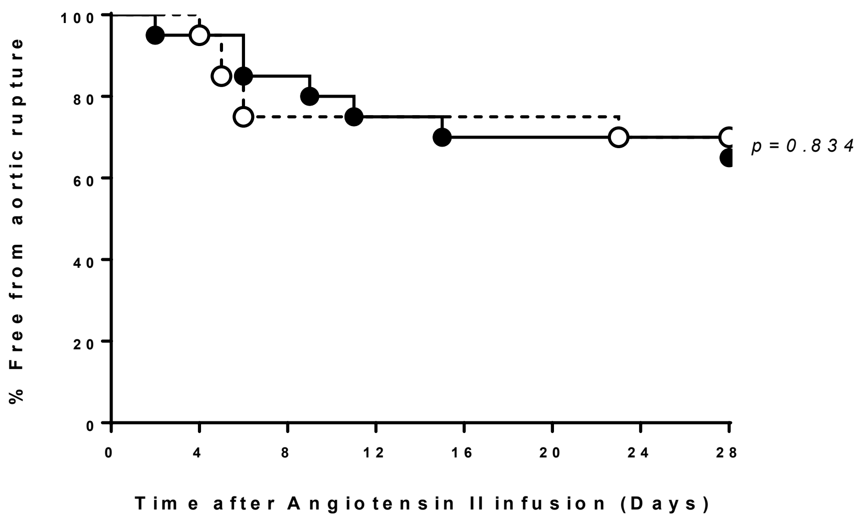

Figure S6. Survival curves of *ApoE$^{-/-}$* mice infused with angiotensin II (AngII) for 28 days and administered rhKAL or vehicle control. Kaplan-Meier curves of survival free from aneurysm rupture in mice administered vehicle control (while circle) or rhKAL (black circle). Data analysed by Mantel-Cox (Log-rank) test (n=20/group).

ApoE $^{-/-}$ **+ AngII + VC (n= 20)**

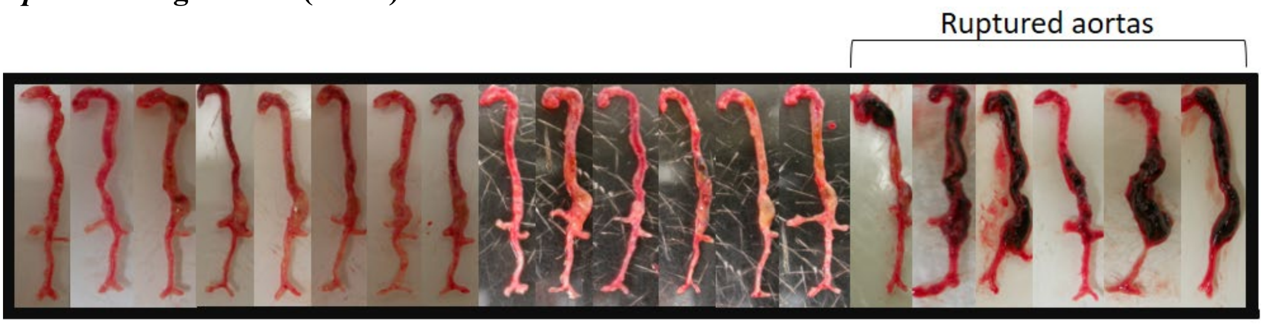

ApoE $^{-/-}$ **+ AngII +** ***rhKAL*** **(n= 20)**

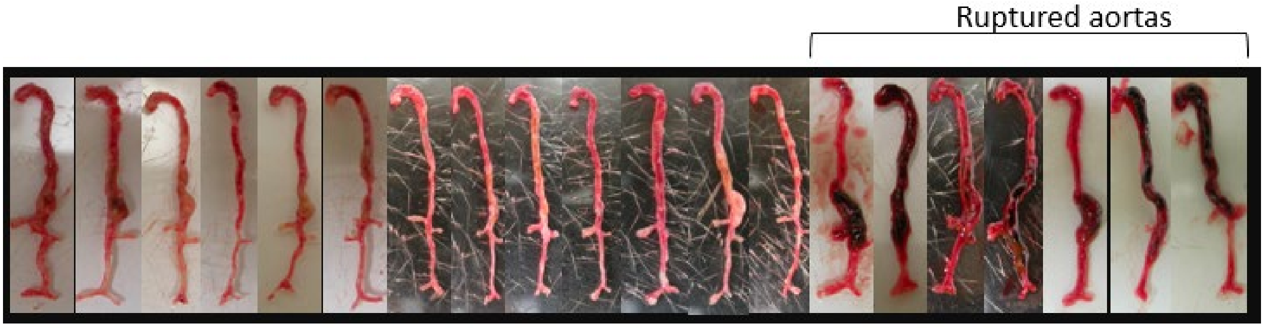

Figure S7: Effect of administration of recombinant human Kallistatin (rhKAL) protein on aortic response to angiotensin II (AngII) infusion for 28 days. Gross morphology of aortas harvested from mice receiving vehicle control (VC) or *rhKAL* while infused with AngII over 28 days. Ruptured aortas are highlighted.

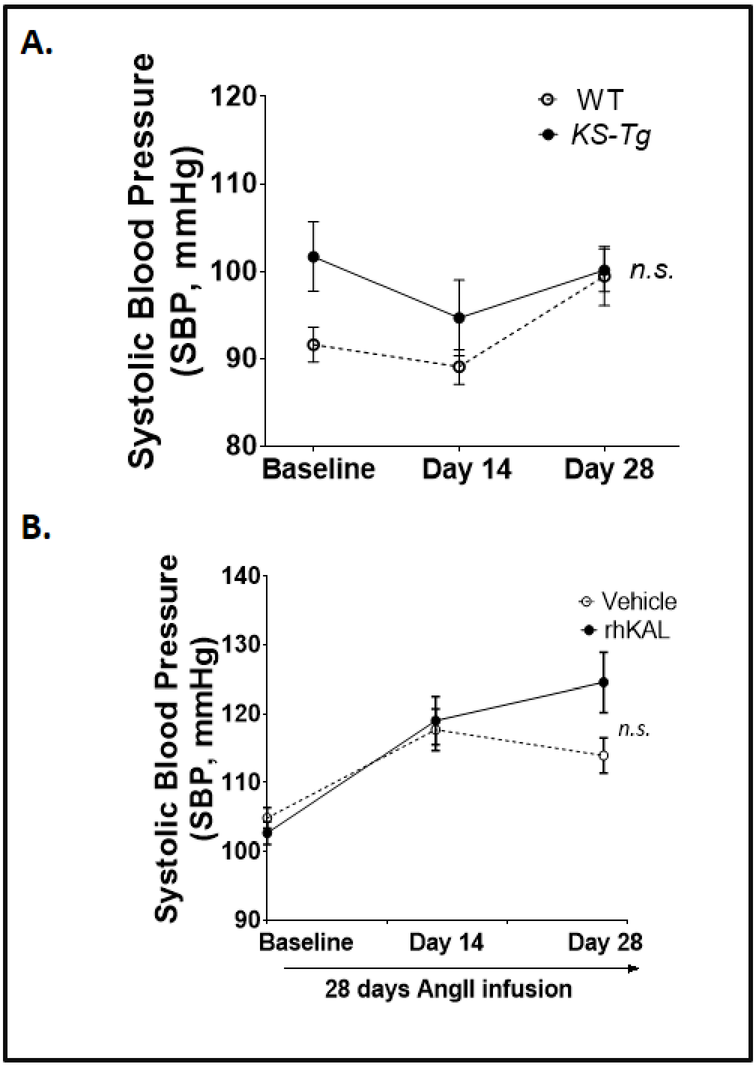

Figure S8: Mean systolic blood pressure measurement in the various study groups. Systolic blood pressure (SBP) was monitored by tail cuff using the CODA machine (Kent Scientific Corporation) following a previously optimised protocol. (A). Quantitative graph showing that compared to the wild type (*WT*) there was no significant difference in mean SPB in Kallistatin transgenic (*KS-Tg*) mice at baseline or at the end of day 28, following $CaPO_4$ administration. (B). Angiotensin II (AngII) infusion for 28 days resulted in a time dependent increase in SBP in both vehicle control *(VC*) and recombinant human KAL (*rhKAL*) administered groups. Compared to control group, the AngII infused rhKAL-administered mice showed a significantly elevated SBP at day 28 post AngII infusion. Statistical analysis by Repeated measures 2-way ANOVA, ns=non-significant.

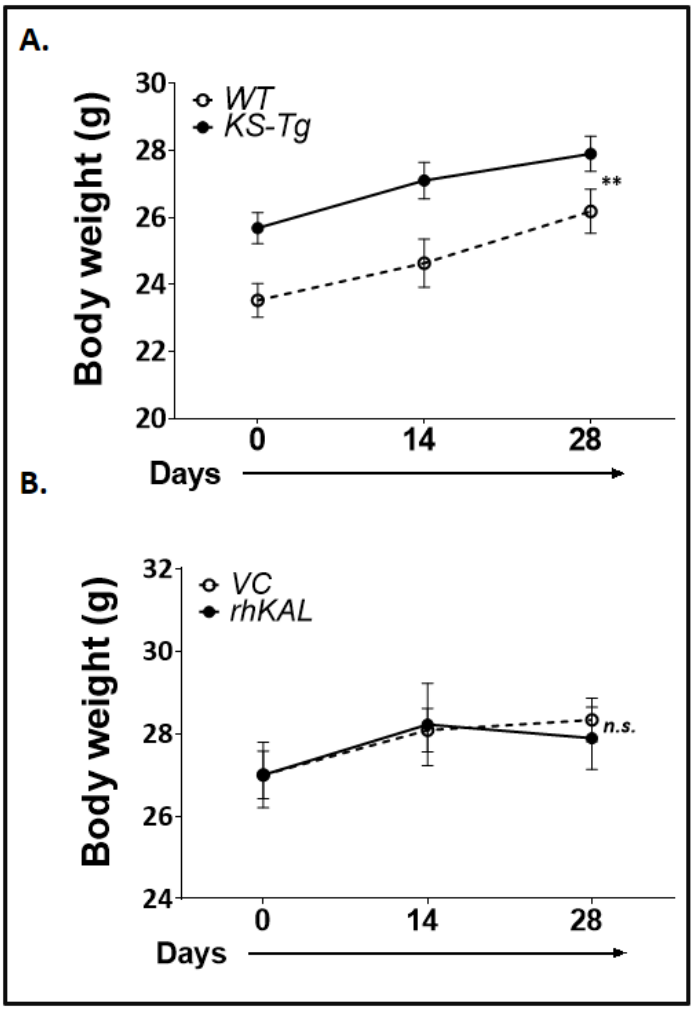

Figure S9: Body weight of mice from the different experimental groups. Body weight was monitored every 14 days. (A). Quantitative graph showing that compared to the wild type (*WT*) Kallistatin transgenic (*KS-Tg*) mice had significantly greater body weight throughout the study period. (B). Angiotensin II (AngII) infusion did not have any influence on body weight in both vehicle control (*VC*) and recombinant human KAL (*rhKAL*) administered groups. Statistical analysis by Repeated measures 2-way ANOVA, **=$P<0.01$.

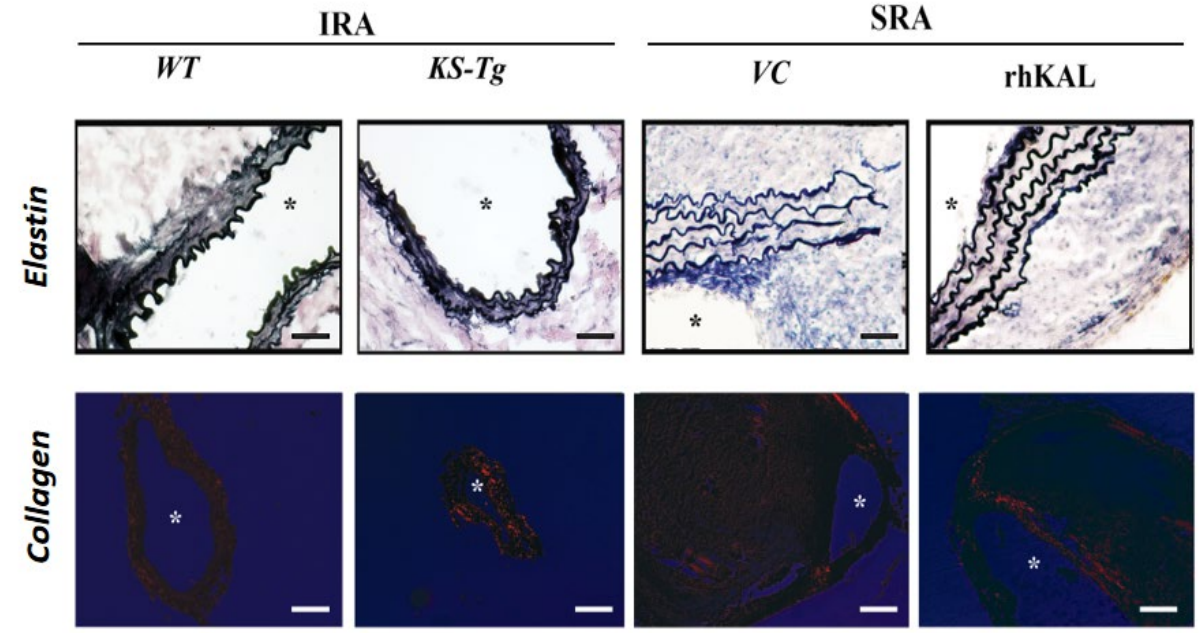

Figure S10: The effect of KAL in the aorta of $CaPO_4$ induced and AngII-induced AAA mouse models. Comparison of medial elastin filament breaks (black structures) within Elastin Verhoeff-Van Gieson (EVG) stained sections of IRA and SRA. Scale bar: 50µm (* Lumen). Polarisation microscopy images of Picrosirius red staining for collagen content. In the $CaPO_4$ model, the IRA of *KS-Tg* mice showed greater collagen birefringence under polarisation compared to wild type mice. Similarly *ApoE*$^{-/-}$ mice receiving rhKAL showed increased collagen birefringence under polarisation compared to controls. Scale bar: 10µm (* Lumen).

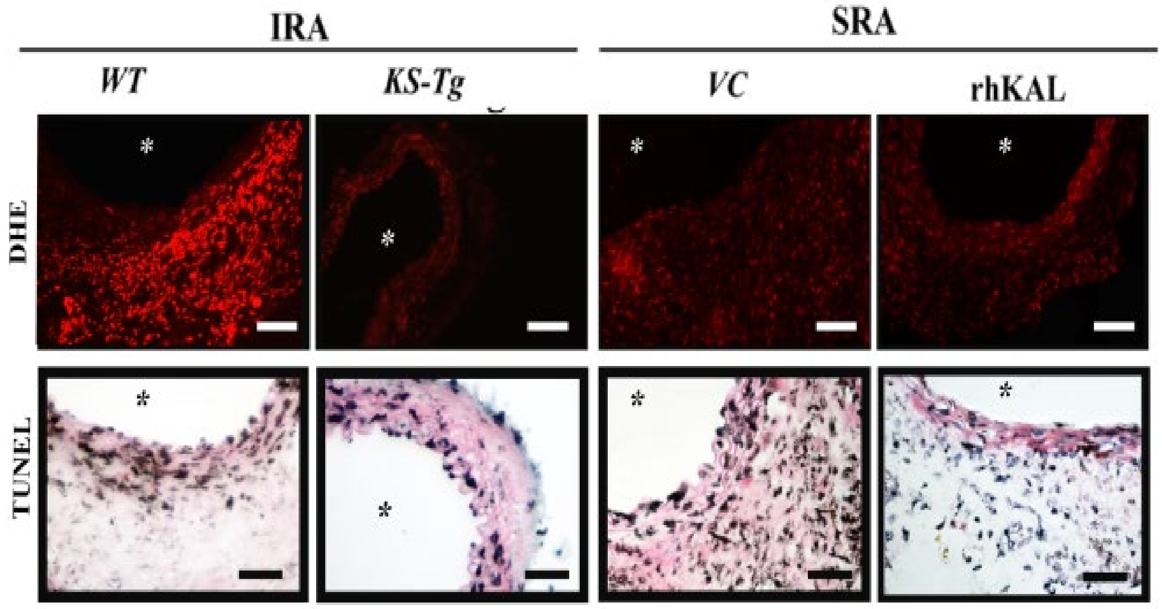

Figure S11: Effect of KAL on oxidative stress and apoptosis in $CaPO_4$ induced and AngII-induced AAA mouse models and *in vitro*. (1) Representative images of *in situ* detection of superoxide by DHE staining in the IRA and SRA sections (Scale bar: 50µm). (2) Second panel showing representative aortic images showing apoptosis as determined by Terminal deoxynucleotidyl transferase dUTP nick end labelling (TUNEL) staining within the aorta of mice receiving $CaPO_4$ or AngII-infused $ApoE^{-/-}$ mice receiving VC or rhKAL (Scale bar: 50µm).

Supplementary Tables.

Table S1.
Comparison of subjects with and without AAA undergoing serum KAL measurement.

**Characteristic**	**AAA**	**No AAA**	***P value***
Number	304	652	
Aortic diameter (mm)	33.40 (31.50-38.70)	21.9 (20.20-23.10)	*<0.001*
Age (years)	71 (68-74)	70 (68-74)	*0.001*
Hypertension	155 (51%)	252 (38.70%)	*<0.001*
Diabetes mellitus	31 (10.20%)	47 (7.20%)	*0.116*
Ever smoker	258 (84.90%)	412 (63.20%)	*<0.001*
CHD	113 (37.20%)	122 (18.70%)	*<0.001*
Previous stroke	22 (7.20%)	28 (4.30%)	*0.057*
WHR	0.97 (0.93-1.01)	0.95 (0.91-0.99)	*<0.001*
Serum creatinine (μM)	95 (82-115)	89 (78-100)	*<0.001*
Serum LDL (mM)	2.60 (2.10-3.20)	2.90 (2.30-3.50)	*<0.001*
Serum HDL (mM)	1.20 (1.10-1.40)	1.40 (1.10-1.60)	*<0.001*
Serum hs-CRP (mg/L)	2.55 (1.37-5.05)	1.77 (0.95-3.65)	*<0.001*
Serum KAL (ng/ml)	14.85 (11.85-18.24)	17.18 (13.76-21.12)	*<0.001*

Nominal variables are presented as numbers (%) and compared by chi-squared. Continuous variables are presented as median (inter-quartile range) and compared by Mann Whitney U test. *Abbreviations:* AAA, Abdominal aortic aneurysm; CHD, Coronary heart disease; WHR, Waist to hip ratio; HDL, High density lipoprotein; LDL, Low density lipoprotein; hsCRP, high sensitivity C-reactive protein; KAL, kallistatin.

Table S2.
Relationship between serum KAL quartiles and AAA growth.

**Serum KAL quartile**	**One**	**Two**	**Three**	**Four**	***P value***
Serum KAL (ng/ml)	<11.96	11.96-14.86	14.86-18	>18	
Number	67	69	68	68	
Age (years)	71 (68-74)	71 (68-75.50)	72 (68-75)	71 (68-75)	*0.920*
Diabetes	5	11	5	7	*0.308*
Hypertension	31	38	35	33	*0.756*
Ever smoker	59	62	55	52	*0.117*
CHD	22	25	25	19	*0.679*
WHR	0.98 (0.93-1.01)	0.96 (0.94-1.01)	0.96 (0.93-1.01)	0.97 (0.93-1.02)	*0.882*
Initial AAA diameter (mm)	34.00 (31.30-41.10)	32.90 (31.45-36.30)	32.65 (30.80-35.25)	32.60 (31.0-34.60)	*0.063*
Serum creatinine (µM)	95 (82-111)	93 (79-112)	90 (76-106)	92.50 (85-114.50)	*0.299*
Serum LDL (mM)	2.60 (2.20-3.20)	2.70 (2.10-3.45)	2.90 (2.13-3.30)	2.70 (2.10-3.40)	*0.895*
Serum HDL (mM)	1.30 (1-1.50)	1.20 (1-1.40)	1.30 (1.10-1.50)	1.20 (1.03-1.40)	*0.392*
Serum hs-CRP (mg/L)	3.64 (1.77-8.63)	2.73 (1.43-6.89)	2.11 (0.99-4.01)	2.05 (1.15-3.57)	*0.001*
Serum KAL (ng/ml)	10.92 (9.37-11.40)	13.41 (12.55-14.12)	16.38 (15.81-17.04)	21.80 (19.57-28.56)	*<0.001*
AAA growth (mm/year)	1.55 (0.93-2.60)	0.92 (0.33-1.91)	0.98 (0.44-1.55)	1.10 (0.47-1.53)	*0.003*
AAA growth (%/year)	4.52 (2.71-7)	2.84 (0.96-5.47)	3.12 (1.26-4.53)	3.12 (1.43-4.52)	*0.007*

Abbreviations: AAA, Abdominal aortic aneurysm; CHD, Coronary heart disease; WHR, Waist to hip ratio; HDL, High density lipoprotein; LDL, Low density lipoprotein; hs-CRP, high sensitivity C-reactive protein; KAL, kallistatin.

Table S3.

Characteristics of AAA patients from whom aneurysmal VSMCs were derived.

AAA-VSMC	Age	Gender	AAA size (cm)
1A	70	Male	53
2A	60	Male	80
3A	67	Male	72
4A	62	Male	90
5A	76	Male	60
6A	77	Female	72

All the samples were obtained from AAA patients that underwent an open surgical repair. AAA size was determined by ultrasound scanning. *Abbreviations*: AAA, abdominal aortic aneurysm; VSMC, vascular smooth muscle cell.

Table S4.

List of human and mouse gene primers used for quantitative real–time PCR assays.

**List of primers used**				
**Human Genes**	**ID**	**Sense Primer**	**Anti-sense Primer**	**Supplier**
MMP-9	*MMP-9*	5'-TATGCCAATAGTCTCCTC-3'	5'-GGCAAGTCTTCCGAGTAG-3'	Premier Biosoft
VEGF	*VEGF*	QT01010184		Qiagen
OPG	*TNFRSF11B*	AATGTGGAATAGATGTTACC	TCTACCAAGACACTAAGC	Premier Biosoft
OPN	*SPP1*	AATGATGAGAGCAATGAG	GTCTACAACCAGCATATC	Premier Biosoft
SIRT1	*SIRT1*	PPH02188A		Qiagen
SERPINA4	*SERPINA4*	ATGGTGCTGGTGAATTAC	GGACTGTTGTGTTCTCATC	Premier Biosoft
GAPDH	*GAPDH*	CTCTGGTAAAGTGGATATTG	GGTGGAATCATATTGGAAC	Premier Biosoft
**Mouse Genes**	**ID**	**Sense Primer**	**Anti-sense Primer**	**Supplier**
Mmp-9	*Mmp-9*	QT00108815		Qiagen
Mmp-2	*Mmp-2*	QT00116116		Qiagen
Vegf	*Vegf*	QT00160769		Qiagen
Opg	*Tnfrsf11b*	AAGATGGCTTCTATTACC	GCTGAAGATAGTCTGTAG	Premier Biosoft
Opn	*Spp1*	ACTCTTCCAAGCAATTCC	GTCTCCATCGTCATCATC	Premier Biosoft
Gapdh	*Gapdh*	QT01658692		Qiagen
